# A genome-wide analysis of carbon catabolite repression in *Schizosaccharomyces pombe*

**DOI:** 10.1186/s12864-019-5602-8

**Published:** 2019-03-29

**Authors:** Dane Vassiliadis, Koon Ho Wong, Alex Andrianopoulos, Brendon J. Monahan

**Affiliations:** 1 Genetics, Genomics & Systems Biology, School of Biosciences, The University of Melbourne, Parkville, Victoria Australia; 2 Commonwealth Scientific and Industrial Research Organisation (CSIRO), Parkville, Victoria Australia; 3 Faculty of Health Sciences, University of Macau, Macau, China; 4 Institute of Translational Medicine, University of Macau, Macau, China; 5 Cancer Therapeutics (CTx), Parkville, Victoria Australia

**Keywords:** *Schizosaccharomyces pombe*, Transcriptional regulation, Carbon metabolism, Carbon catabolite repression, Scr1, Tup11, Rst2, RNA-seq, ChIP-seq

## Abstract

**Background:**

Optimal glucose metabolism is central to the growth and development of cells. In microbial eukaryotes, carbon catabolite repression (CCR) mediates the preferential utilization of glucose, primarily by repressing alternate carbon source utilization. In fission yeast, CCR is mediated by transcriptional repressors Scr1 and the Tup/Ssn6 complex, with the Rst2 transcription factor important for activation of gluconeogenesis and sexual differentiation genes upon derepression. Through genetic and genome-wide methods, this study aimed to comprehensively characterize CCR in fission yeast by identifying the genes and biological processes that are regulated by Scr1, Tup/Ssn6 and Rst2, the core CCR machinery.

**Results:**

The transcriptional response of fission yeast to glucose-sufficient or glucose-deficient growth conditions in wild type and CCR mutant cells was determined by RNA-seq and ChIP-seq. Scr1 was found to regulate genes involved in carbon metabolism, hexose uptake, gluconeogenesis and the TCA cycle. Surprisingly, a role for Scr1 in the suppression of sexual differentiation was also identified, as homothallic scr1 deletion mutants showed ectopic meiosis in carbon and nitrogen rich conditions. ChIP-seq characterised the targets of Tup/Ssn6 and Rst2 identifying regulatory roles within and independent of CCR. Finally, a subset of genes bound by all three factors was identified, implying that regulation of certain loci may be modulated in a competitive fashion between the Scr1, Tup/Ssn6 repressors and the Rst2 activator.

**Conclusions:**

By identifying the genes directly and indirectly regulated by Scr1, Tup/Ssn6 and Rst2, this study comprehensively defined the gene regulatory networks of CCR in fission yeast and revealed the transcriptional complexities governing this system.

**Electronic supplementary material:**

The online version of this article (10.1186/s12864-019-5602-8) contains supplementary material, which is available to authorized users.

## Background

Optimal carbon metabolism is vital to energy homeostasis and directly impacts the growth and development of cells. Glucose is a preferred source of carbon for most organisms and transcriptional or post-translational regulatory mechanisms have evolved to modulate gene expression to utilize available glucose in preference to other carbon sources, allowing cells to adapt to changing environmental carbon conditions. The transcriptional regulatory mechanism enforcing this preferential glucose utilization is referred to as carbon catabolite repression (CCR). CCR regulates several groups of genes including those required for sugar transport, carbon metabolism and cell proliferation, and dysregulation of these pathways can have severe physiological or pathological consequences [1–3].

In the fission yeast, *Schizosaccharomyces pombe*, CCR is mediated by Scr1, a conserved C_2_H_2_ zinc finger transcriptional repressor orthologous to the well characterized *Saccharomyces cerevisiae* Mig1 and *Aspergillus nidulans* CreA [4]. Mig1 and CreA possess strongly conserved zinc finger domains that bind similar DNA motifs, 5′-SYGGGG-3′ and 5′-SYGGRG-3′ respectively (Additional file 1: Figure S1) [5, 6]. At chromatin, CCR transcription factors co-localise with additional transcriptional machinery, such as the conserved Tup/Ssn6 complex, which is required to establish full transcriptional repression of CCR regulated genes [7–9]. In *S. cerevisiae*, various models of Tup/Ssn6 action have been proposed, ranging from the additional recruitment of chromatin modifying or remodeling machinery, to interference of RNA Pol II and Mediator [10–12]. The local glucose concentration tightly regulates the function and localisation of Scr1/Mig1/CreA and their interaction with Tup/Ssn6 [13–15]. In *S. pombe* and *S. cerevisiae*, this occurs via AMP-activated protein kinase (AMPK) mediated phosphorylation of Scr1/Mig1 upon glucose depletion, which dissociates these transcription factors from chromatin and triggers their nuclear export [16, 17].

Expression profiling analyses have revealed a diverse set of biological processes that are regulated by CCR in *S. cerevisiae*, *A. nidulans* and *Trichoderma reesei* [18–22]. However, in *S. pombe*, Scr1-mediated CCR has been shown to regulate just four genes: *inv1*^+^ (invertase), *gld1*^+^ (glycerol de-hydrogenase), *fbp1*^+^ (fructose bis-phosphatase), and *ght5*^+^ (transmembrane hexose transporter) [4, 23–25]. Loss of *scr1*^+^ function results in the loss of repression and the inappropriate expression of *inv1*^+^, *gld1*^+^ and *ght5*^+^ in the presence of glucose, indicating that Scr1 is chiefly responsible for their regulation in glucose-sufficient conditions. In contrast, loss of *scr1*^+^ results in poor *fbp1*^+^ upregulation presumably because *fbp1*^+^ requires active induction by Rst2 in the absence of repression via Scr1 [13]. Along with Scr1, the two *S. pombe* Tup1 orthologues, Tup11 and Tup12 are required for full repression of *fbp1*^+^ in glucose-replete conditions [13, 26]. In the absence of glucose, Scr1 is hyper-phosphorylated by the AMPK, Ssp2, and exported from the nucleus [13, 17]. This process has been thoroughly examined at the *fbp1*^+^ locus, whereby Scr1 is replaced at the *fbp1*^*+*^ promoter by the transcriptional activator Rst2, which induces *fbp1*^+^ expression to drive gluconeogenesis via potential modulation of Tup/Ssn6 function [13]. Rst2 contains a similar C_2_H_2_ zinc finger to Scr1, *S. cerevisiae* Mig1, and other CCR repressors (Additional file 1: Figure S1), and binds the DNA motif 5′-CCCCTC-3′, the complement of which is strikingly similar to the DNA binding motifs of *S. cerevisiae* Mig1 and *A. nidulans* CreA [27]. This suggests that, on a genome-wide level, CCR regulated genes may be toggled between repression and activation by opposing roles of Scr1 and Rst2.

Fungal systems, particularly the yeasts *S. pombe* and *S. cerevisiae*, are of major industrial importance in the production of consumable products, bio-fuels & pharmaceuticals [28–30]. These industrial processes rely on efficient utilization of the intrinsic carbon metabolism and fermentative capabilities of these organisms, and so, deepening our understanding of CCR and its governance over these metabolic processes will inform the development of novel strains which exhibit improved industrial performance phenotypes. To date, the gene regulatory networks controlled by CCR in *S. pombe* remain largely undefined. Using genetic and genome-wide methods, this study aimed to comprehensively characterize CCR in fission yeast by identifying the genes and biological processes regulated by Scr1, Tup/Ssn6 and Rst2. In the presence of glucose, *scr1*^*−*^ loss of function mutants showed derepression of genes involved in carbon source metabolism, hexose uptake, and gluconeogenesis. In addition, a role for CCR in the repression of sexual differentiation, a key indicator of nutrient stress in *S. pombe*, was identified. ChIP-seq analysis of Scr1, Tup11 and Rst2 localisation revealed striking co-occupancy of Scr1-Tup11 or Rst2-Tup11 at gene promoters in glucose-sufficient or glucose-deficient conditions respectively. Moreover, in glucose-sufficient conditions, co-occupancy of Scr1 and Rst2 was observed at a subset of key carbon metabolism genes also bound by Tup11, suggesting these two transcription factors may compete for binding to these target loci and that this may influence the transcriptional regulatory function of Tup/Ssn6. Overall these findings define several biological roles for the *S. pombe* CCR transcriptional machinery, paving the way for further in-depth functional characterisation of the *S. pombe* glucose repression network.

## Results

### Scr1 regulates a core subset of glucose-regulated genes in *S. pombe*

To identify genes with altered expression in response to glucose availability in *S. pombe*, RNA sequencing (RNA-seq) was performed on wild type cells from glucose-sufficient (YES 3% glucose), sucrose (YES 3% sucrose), glucose-deficient (YES 3% glycerol + 0.1% glucose), or glucose-starved (YES 3% glycerol) growth conditions (Additional file 1: Figure S2A). Pairwise comparison of all conditions revealed 2374 genes (approximately 46% of all *S. pombe* genes) responsive to glucose, which is consistent with the proportion of glucose-responsive genes identified in similar studies in *S. cerevisiae* [31–33] (Additional file 2: Table S1). We also identified 1825 ncRNAs, tRNAs or other small RNAs, which were differentially expressed in at least one pairwise comparison between conditions (Additional file 3: Table S2). Most (1749, 73.6%) of these 2374 genes were differentially expressed in the glucose-starved condition compared to glucose or sucrose (Additional file 2: Table S1), suggesting that glycerol as a sole carbon source elicits an extreme carbon starvation response in *S. pombe*, leading to a significant reprogramming of transcriptional activity (Additional file 1: Figure S2B). In contrast, the glucose and sucrose conditions elicited similar transcriptional responses (Additional file 1: Figure S2C). Gene ontology (GO) analysis highlighted carbon metabolism pathways, including the tricarboxylc citric-acid (TCA) cycle and the pentose phosphate shunt as significantly upregulated in either glucose-deficient or glucose-starved conditions (Additional file 1: Figure S2D&E). Conjugation and response to pheromone genes were upregulated specifically in the glucose-deficient condition indicating induction of the sexual differentiation program consistent with previous work [34], while cellular autophagy genes were upregulated in the glucose-starved condition reflecting a lack of growth in carbon-starved media [23]. We examined key carbon metabolism pathways in further detail, confirming widespread upregulation of almost all carbon metabolism genes examined in glucose-deficient conditions, while glycolysis and fermentation genes were repressed under glucose-starved conditions (Additional file 1: Figure S3).

The induction of carbon metabolism transcriptional signatures in wild type cells cultured in the absence of glucose led us to study factors which may maintain repression of these pathways in glucose-replete conditions. To study the transcriptional consequences of *scr1*^*−*^ loss of function on the *S. pombe* transcriptome, diploid *S. pombe scr1*^+^/*scr1*Δ heterozygotes were generated, and sporulated to isolate haploid *scr1*Δ cells (Additional file 1: Figure S4A). A complete *scr1*Δ deletion has not been described previously, nor is it present in *S. pombe* whole genome deletion collections [35, 36]. However the growth characteristics of our *scr1*Δ deletion strain was no different to previously described *scr1*::*ura4*^*+*^ disruption strains on YES glucose medium [4, 13, 24] suggesting complete loss of *scr1*^+^ does not impair *S. pombe* viability under such conditions (Additional file 1: Figure S4B). To identify Scr1 regulated genes we sequenced RNA from *scr1*Δ and *scr1*::*ura4*^*+*^ cells shifted to either glucose or sucrose conditions (Additional file 1: Figure S2A). Expression of protein-coding genes in *scr1*Δ and *scr1*::*ura4*^*+*^ strains in either condition were strongly correlated (R^2^ ~ 0.97), suggesting minimal differences between the transcriptional profiles of the two strains in glucose and sucrose conditions (Additional file 1: Figure S4C). Therefore for all further analyses, the *scr1*Δ and *scr1*::*ura4*^*+*^ RNA-seq samples were analysed as a single condition (hereafter “*scr1*^−^ mutant”).

**Fig. 1:**
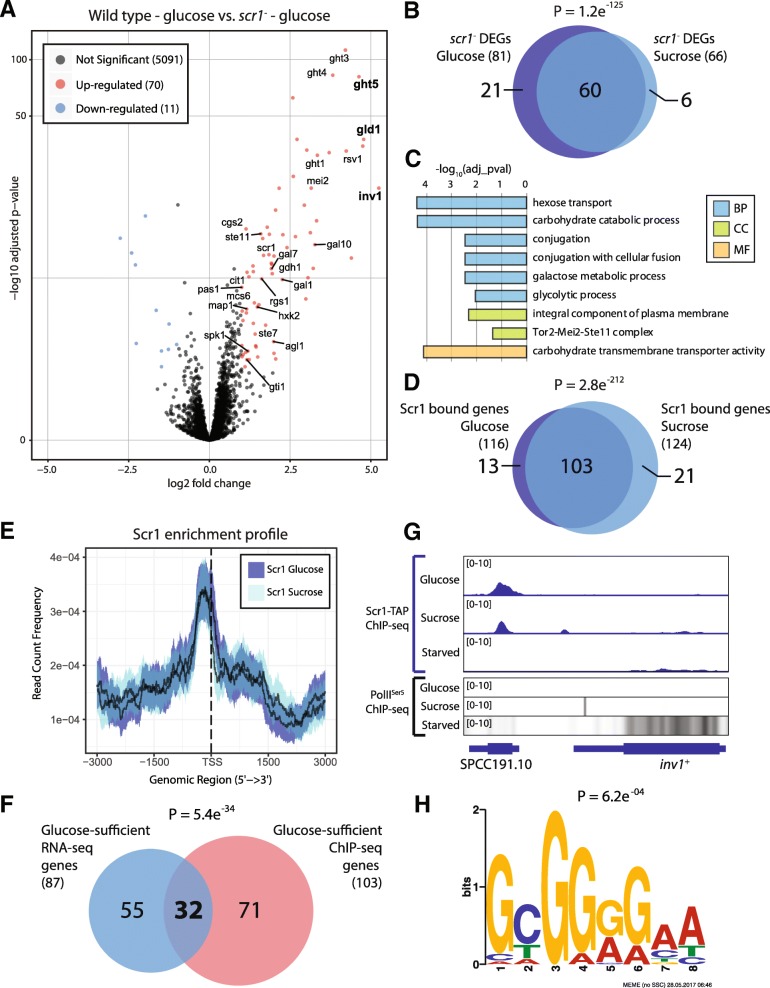
Scr1 regulates a core subset of genes in *S. pombe*. **a** Volcano plot of log2 fold change (x-axis) vs -log10 adjusted *p*-value (y-axis, log scale) for *S. pombe* protein-coding genes in the *scr1*^−^ mutant background vs. wild type for the glucose condition. Down-regulated (blue), and up-regulated (red) points indicate genes that met both log2 fold change (− 1 > Log_2_FC > 1) and adjusted p-value (P_adj_ < 0.05) thresholds. Genes of interest are labelled. Known Scr1 regulated genes are shown in bold. **b** Overlap of genes differentially expressed in the *scr1*^−^ mutant vs wild type *S. pombe* on glucose vs sucrose. **c** GO enrichment analysis of 87 genes differentially expressed in the *scr1*^−^ mutant in glucose and sucrose. BP = Biological process, CC = cellular component, MF = molecular function. **d** Overlap of protein-coding genes annotated to Scr1 ChIP-seq peaks in glucose and sucrose conditions. **e** Enrichment profile of Scr1 surrounding *S. pombe* gene transcription start sites (TSS) in glucose and sucrose conditions. Solid line indicates the mean while the coloured region defines a 95% confidence interval. **f** Overlap of differentially expressed genes in *scr1*^−^ mutant RNA-seq vs a consensus set of protein-coding genes enriched for Scr1 in glucose and sucrose. **g** Genome browser visualization of Scr1 and RNA PolII^Ser5^ (as heatmap) enrichment at the *inv1*^+^ locus. **h** Top hit from MEME-ChIP motif analysis of the peak regions from the 32 genes both bound by Scr1 and differentially expressed in the *scr1*^−^ mutant in the glucose condition. Venn diagram *p*-values were calculated using the hypergeometric probability distribution

Eighty-one genes (~ 1.5% of the *S. pombe* genome) were significantly differentially expressed in the *scr1*^*−*^ mutant in the glucose condition compared to wild type *S. pombe* (Fig. 1a). Of these, 70 (86%) were upregulated, consistent with the primary role of Scr1 as a transcriptional repressor (Additional file 4: Table S3). Among the upregulated genes were the previously reported targets of Scr1: *inv1*^+^*, gld1*^+^ and *ght5*^+^. [4, 23, 25]. An analogous pattern was observed for the sucrose condition (Additional file 4: Table S3), indicating that Scr1 regulates a largely similar set of genes in glucose and sucrose (Fig. 1b). By combining our analysis of both conditions, 87 differentially expressed genes (DEGs) regulated by Scr1 in glucose and sucrose (hereafter “glucose-sufficient”) conditions were identified (Additional file 5: Table S4). GO analysis of this gene set identified canonical CCR pathways but also unexpected categories including galactose metabolism, conjugation and Tor2-Mei2-Ste11 complex (Fig. 1c).

We next performed ChIP-seq to identify genes directly bound by Scr1. As no Scr1 specific antibody was available, a tandem affinity purification (TAP)-tagged allele of *scr1* was generated at the endogenous locus using an allelic replacement strategy (Additional file 1: Figure S5). Scr1 chromosomal occupancy was examined in glucose, sucrose, and glucose-starved conditions using identical culture methods to previous RNA-seq experiments (Additional file 1: Figure S2A). In addition, ChIP-seq of RNA Polymerase II (RNA PolII^Ser5^) was performed from the same chromatin samples to examine active transcriptional elongation in each condition. Scr1-TAP was significantly enriched upstream of 116 and 124 protein-coding genes in the glucose and sucrose conditions respectively (Additional file 6: Table S5). No significant enrichment was detected for Scr1 in the glucose-starved condition, consistent with Scr1 being excluded from the nucleus in the absence of glucose [13, 25]. An additional 24 and 31 ncRNA, or other small RNA, encoding loci were annotated to regions of Scr1 enrichment in glucose and sucrose respectively (Additional file 6: Table S5). Consistent with the RNA-seq data, a significant overlap between the set of protein-coding genes bound by Scr1 in glucose and sucrose was observed (Fig. 1d). In both conditions, Scr1 was predominantly enriched upstream of gene transcription start sites (TSS) (Fig. 1e). Unexpectedly, just 32 of the 87 (37%) “glucose-sufficient” Scr1-regulated genes defined via the RNA-seq analysis were also directly bound by Scr1 in these conditions (Fig. 1f). Among this subset were Scr1 regulated genes, *inv1*^+^ (Fig. 1g)*, gld1*^+^ and *ght5*^+^ [4, 23, 25], and also *scr1*^+^ (Additional file 1: Figure S6A) indicating that Scr1 has autoregulatory capability. Enrichment of RNA PolII^Ser5^ was consistent with the repression of these genes in glucose and sucrose and their activation in glucose-starved conditions (Fig. 1g, Additional file 1: Figure S6B). We termed these 32 core Scr1 targets as “Scr1-dependent” genes (Additional file 6: Table S5). Fifty-five additional genes that were differentially expressed in the *scr1*^−^ RNA-seq, but not bound by Scr1, were defined as indirectly regulated Scr1 target genes (“Scr1-indirect target genes”, Additional file 6: Table S5), and a further 71 genes bound by Scr1, but not significantly differentially expressed in the *scr1*^−^ background, were defined as Scr1 direct target genes (“Scr1-direct target genes”, Additional file 6: Table S5). MEME-ChIP [37, 38] analysis of the Scr1 bound regions upstream of the “Scr1-dependent” genes identified a 5′ – SYGGRG – 3′ motif, identical to the *A. nidulans* CreA motif, significantly enriched and found in 31/32 gene promoter regions (Fig. 1h, Additional file 1: Figure S7).

**Fig. 2:**
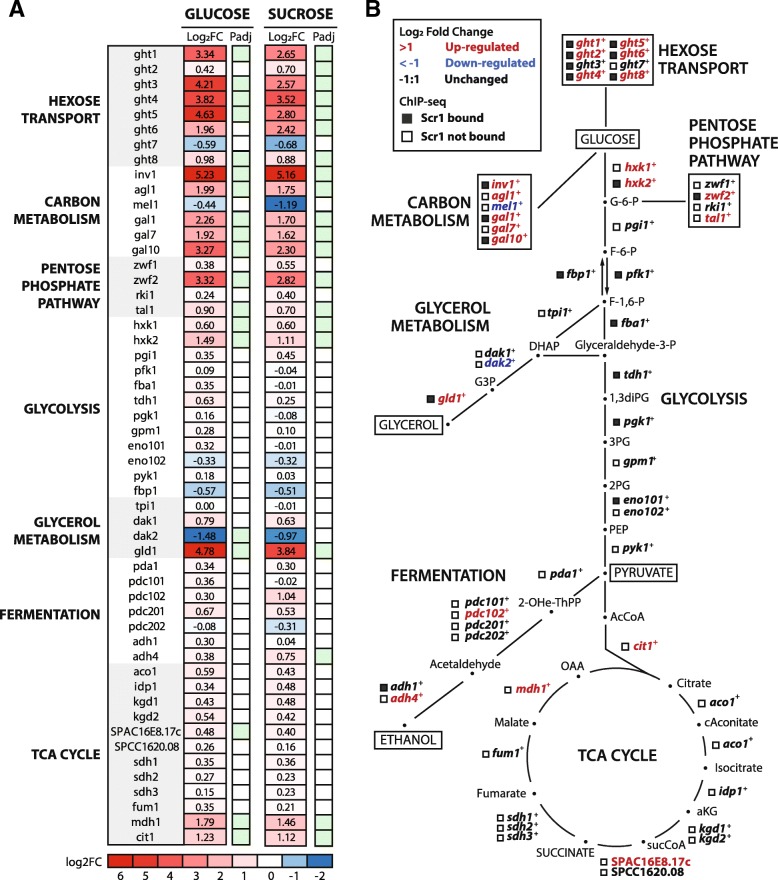
Scr1 plays a key role in the regulation of carbon metabolism. **a** Heatmap of Log_2_FC for selected genes involved in carbon metabolism pathways for the *scr1*^−^ mutant vs wild type *S. pombe* in the glucose and sucrose conditions. Green boxes indicate genes significant at an FDR < 0.05. **b** Pathway diagram of carbon metabolism genes from **a** colored according to the key. Boxes indicate whether Scr1 was bound (filled) or not bound (open) at that gene promoter in the consensus set of protein coding genes bound by Scr1 in glucose or sucrose conditions (Additional file 6: Table S5)

The enrichment of carbon metabolism pathways amongst the set of Scr1 regulated genes prompted us to directly examine the transcriptional response of carbon metabolism genes to *scr1*^−^ loss of function. Most genes showed either unchanged or increased expression in the *scr1*^−^ mutant background relative to wild type in the glucose and sucrose conditions (Fig. 2a). Interestingly, the glycolysis/gluconeogenesis genes *pfk1*^+^, *fbp1*^+^, *fba1*^+^, *tdh1*^+^, *pgk1*^+^, and *eno101*^+^ were bound by Scr1, but showed no significant change in expression in the *scr1*^−^ mutant background suggesting other factors are also involved in their regulation (Fig. 2b). We examined the set of 127 Scr1 ChIP-seq peaks annotated to protein-coding genes or non-coding regions in glucose and sucrose conditions and identified two additional enriched motifs, the HAP complex CCAAT box (5′ – CCAATC – 3′, *P* = 1.0xE^− 07^), and an ATF/CREB-like motif (5′ – ACATCA – 3′, *P* = 1.03E^− 03^). These results suggest that the HAP complex and/or ATF/CREB transcription factors may regulate these genes alongside Scr1, as was shown previously for *fbp1*^+^ [24]. Together, these results define the glucose-responsive gene regulatory network in *S. pombe* and the role of Scr1-mediated CCR within this process.

### Loss of *scr1*^+^ function results in ectopic meiosis

**Fig. 3:**
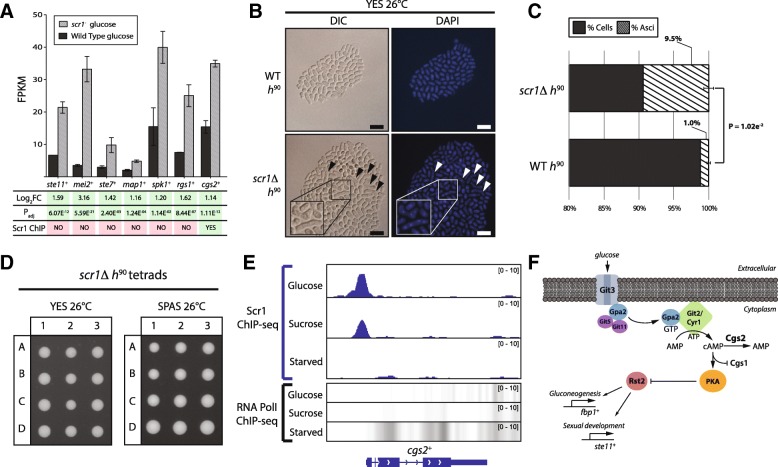
Loss of *scr1*^+^ results in ectopic meiosis under carbon and nitrogen replete conditions. **a** Histogram of wild type and *scr1*^−^ mutant expression for meiosis genes upregulated in the glucose condition in the *scr1*^−^ background. Green/red shading indicates pass/fail of Log_2_FC, P_adj_ RNA-seq thresholds and Scr1 enrichment by ChIP-seq (Scr1 ChIP). Only *cgs2*^+^ appears both directly regulated by Scr1 and significantly upregulated in the *scr1*^−^ background. **b** DIC and DAPI microscopy of homothallic wild type (WT *h*^90^) and *scr1*Δ (*scr1*Δ *h*^90^) *S. pombe* cells grown on YES at 26 °C. Arrows and inset panel indicate meiotic asci. Scale bars = 20 μm. **c** Quantification of the proportion of meiotic asci in the *scr1*Δ *h*^90^ and WT *h*^90^ populations out of a total of at least 1000 cells. *P*-value reflects a two-tailed students T-test assuming unequal variance from three biological replicates. **d** Representative dissection of *scr1*Δ *h*^90^ meiotic tetrads under normal (SPAS 26 °C) or ectopic (YES 26 °C) mating conditions. **e** Genome browser visualization of Scr1 and RNA PolII^Ser5^ enrichment at the *cgs2*^+^ locus in the glucose, sucrose and glucose-starved “Starved” conditions. **f** Overview of the *S. pombe* Git3/Gpa2 glucose sensing pathway

An interesting observation from the RNA-seq analysis was the upregulation of multiple meiosis and sexual differentiation genes in the *scr1*^−^ mutant compared to wild type under nitrogen and glucose-sufficient conditions where mating is usually repressed (Fig. 3a). To investigate whether *scr1*Δ deletion and the subsequent upregulation of *ste11*^+^, *mei2*^+^ and other sexual differentiation genes could induce ectopic mating, *scr1*Δ *h*^90^ and wild type *h*^90^ strains were inoculated onto YES 3% (*w*/*v*) glucose or sporulation agar (SPAS) plates and monitored for sexual differentiation. Both strains formed normal asci and viable offspring when cultured on SPAS at 26 °C (normal mating conditions) confirming no deficiency in meiosis (Additional file 1: Figure S8). Remarkably, clear formation of zygotic asci in the *scr1*Δ *h*^90^ population on YES medium was observed (Fig. 3b) at a frequency of 9.5% compared to 1.0% for wild type *h*^90^ *S. pombe* (Fig. 3c). Dissection of zygotic *scr1*Δ *h*^90^ tetrads revealed no viability defects in the resulting progeny suggesting that meiosis had completed successfully (Fig. 3d). Overall, these results suggest that glucose is an important factor regulating mating in *S. pombe*, and that Scr1 is required to repress sexual differentiation under glucose-sufficient conditions. Interestingly, ChIP-seq data showed that *ste11*^+^ and the other sexual differentiation genes upregulated in the *scr1*^*−*^ mutant background were not bound by Scr1 in glucose-sufficient conditions (Fig. 3a). An exception was the *cgs2*^+^ gene, encoding a cAMP-specific phosphodiesterase, which was both bound by Scr1 (Fig. 3e) and significantly upregulated in the *scr1*^*−*^ mutant background in glucose-sufficient conditions (Fig. 3a, Additional file 4: Table S3). It has been shown that upregulation of *cgs2*^+^, in response to nutrient limitation results in a reduction in cellular cAMP levels, subsequent inactivation of protein kinase A (PKA) via Cgs1 and induction of *ste11*^+^ by Rst2 [39, 40] (Fig. 3f). Therefore, derepression of *cgs2*^+^ in the *scr1*Δ *h*^90^ mutant background in carbon and nitrogen replete conditions may drive this ectopic meiosis phenotype.

### Scr1 and Tup/Ssn6 co-localise at the promoter of CCR regulated genes

**Fig. 4:**
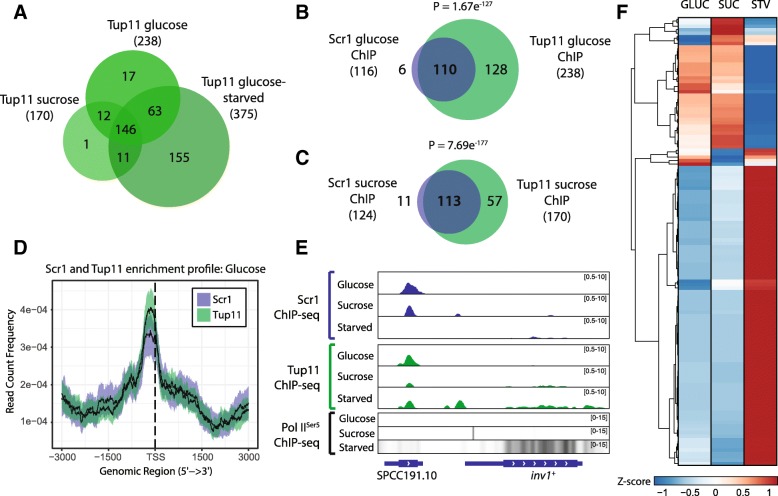
Tup11 and Scr1 co-localise at gene promoters. **a** Overlap of protein-coding genes annotated to Tup11 ChIP-seq peaks in the glucose, sucrose and glucose-starved conditions. Overlap of Scr1 and Tup11 bound protein-coding genes in glucose (**b**) and sucrose (**c**) conditions. **d** Enrichment profiles of Scr1 and Tup11 surrounding *S. pombe* gene transcription start sites in glucose and sucrose conditions. Solid line indicates the mean while the coloured region defines a 95% confidence interval. **e** Genome browser visualization of Scr1, Tup11 and RNA PolII^Ser5^ enrichment at the *inv1*^+^ locus in the glucose, sucrose and glucose-starved (Starved) conditions. **f** Heatmap of gene expression in wild type cells for 130 protein-coding genes bound by both Scr1 and Tup11 in glucose or sucrose. Venn diagram *P*-values calculated using the hypergeometric probability distribution

Scr1-mediated CCR requires the recruitment and function of the Tup/Ssn6 complex for maximal repression [24]. In *S. pombe*, Tup11 and Tup12 appear functionally redundant and interact with Ssn6 to form the stable Tup/Ssn6 protein complex, generally comprised of all three proteins [41, 42]. ChIP-seq of Tup11 as a proxy for localisation of the Tup/Ssn6 complex at chromatin, was performed using a Tup11-TAP tagged strain and the same growth conditions as for the Scr1-TAP ChIP-seq. Tup11 was bound at 238, 170 and 375 protein-coding genes in glucose, sucrose, and glucose-starved conditions respectively, with an additional 60, 42 and 85 ncRNAs, or other small RNA encoding loci also bound in each condition (Fig. 4a, Additional file 7: Table S6). Almost all Scr1 bound genes were also bound by Tup11 (Fig. 4b-c) and their binding overlapped upstream of the TSS of genes (Fig. 4d). Co-localisation of the two factors was observed at multiple loci known to be Scr1 and Tup11 regulated, including *inv1*^+^ (Fig. 4e), *ght5*^+^ and *gld1*^+^ (Additional file 1: Figure S9). Moreover, association of Scr1 and Tup11 at loci in glucose or sucrose was predominantly associated with reduced expression in wild type cells compared to glucose-starved conditions (Fig. 4f).

We then investigated whether Scr1 physically interacts with the Tup/Ssn6 complex by performing Scr1 or Tup11 tandem affinity purifications (TAP) [43] and monitoring the co-immunoprecipitation of FLAG-tagged Tup11 or Scr1, respectively. To enable this analysis, *scr1*-FLAG, *tup11*-TAP and *tup11*-FLAG, *scr1*-TAP strains were generated by crossing the appropriate FLAG and TAP tagged strains. Tup11-FLAG was co-purified alongside Scr1-TAP confirming that Tup11 does interact with Scr1 (Additional file 1: Figure S10). Scr1-FLAG also co-purified with Tup11-TAP but could only be detected after the first affinity purification. Mass spectrometry of the Tup11-TAP samples revealed both Tup12 and Ssn6 components of the complex but did not detect Scr1 (data not shown). Conversely Tup/Ssn6 components were not detected in Scr1-TAP samples, suggesting that while Scr1 and Tup11 interact, they do not exist in a stable stoichiometric protein complex. Together, these findings confirm that the Tup/Ssn6 complex physically co-localises with Scr1 at chromatin in glucose-sufficient conditions to enforce the repression of CCR target genes.

### Tup11 and Rst2 co-localise at CCR genes and actively transcribed loci in glucose-starved conditions

**Fig. 5:**
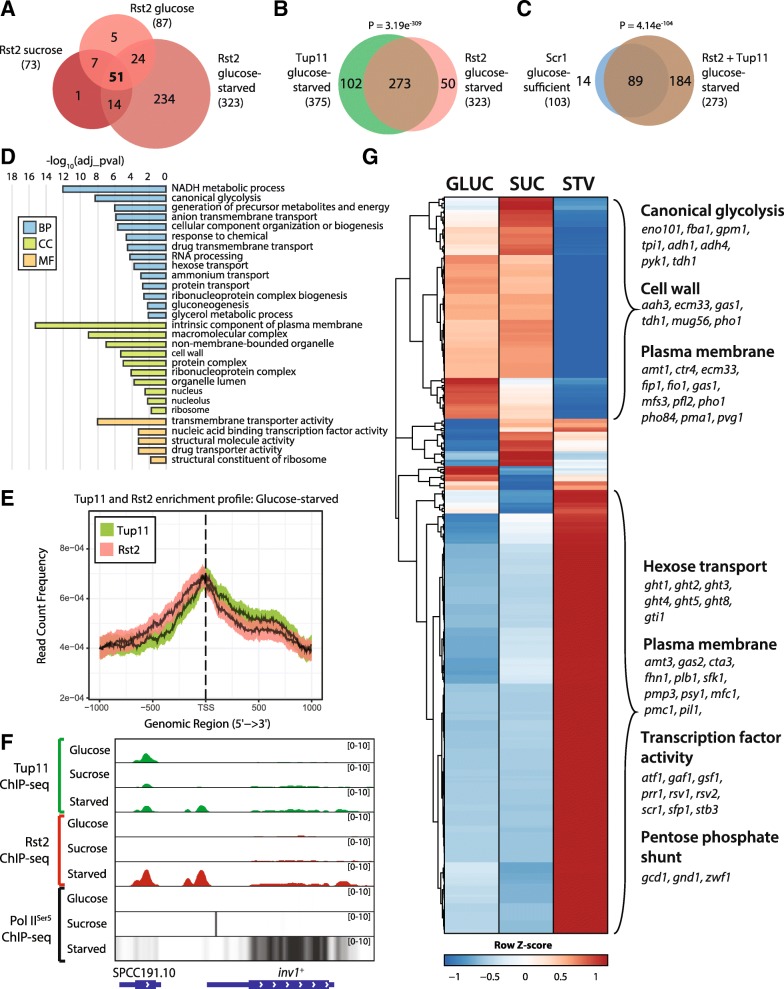
Tup11 co-localises with Rst2 at gene promoters in glucose-starved conditions. **a** Overlap of protein-coding genes annotated to Rst2 ChIP-seq peaks in the glucose, sucrose and glucose-starved conditions. **b** Overlap of protein-coding genes annotated to Tup11 or Rst2 ChIP-seq peaks in the glucose-starved condition. **c** Overlap of protein-coding genes enriched for Scr1 in glucose or sucrose conditions and protein-coding genes enriched for Tup11 and Rst2 in glucose-starved conditions **d** GO terms enriched in the subsets of protein-coding genes bound by Rst2 and Tup11 in glucose-starved conditions. **e** Enrichment profiles of Tup11 and Rst2 in the glucose-starved condition. Solid line indicates the mean while the coloured region indicates a 95% confidence interval. **f** Genome browser view of Tup11, Rst2 and RNA Pol II^Ser5^ ChIP-seq enrichment at the *inv1*^+^ locus in glucose, sucrose and glucose-starved (Starved) conditions. The black arrows indicate significant regions of Tup11 and Rst2 enrichment. **g** Heatmap of gene expression in wild type cells grown in glucose, sucrose and glucose-starved conditions for protein-coding and non-coding loci enriched for both Tup11 and Rst2 in glucose-starved conditions. Enriched GO categories and representative genes are shown to the right for specific clusters within the heatmap. Venn diagram *P*-values calculated using the hypergeometric probability distribution

The Rst2 transcriptional activator associates with Tup/Ssn6 upstream of *fbp1*^+^ and is required for expression in glucose-deficient conditions [13, 27]. Given the widespread remodeling of transcription in the glucose-starved condition, including induction of carbon metabolism and gluconeogenesis genes (Additional file 1: Figure S2-S3), we investigated potential roles for Rst2-Tup/Ssn6 co-localisation in this process. A C-terminally TAP-tagged Rst2 (Rst2-TAP) expressing strain was generated and confirmed by western blot (Additional file 1: Figure S11A), and Rst2 chromosomal occupancy was examined in glucose, sucrose, and glucose-starved conditions. Overall, 87, 73, and 323 protein-coding genes were bound by Rst2 in glucose, sucrose, and glucose-starved conditions respectively, with 35, 27 and 90 ncRNAs, or other small RNA encoding loci also bound by Rst2 in each condition (Additional file 8: Table S7). The majority of Rst2 bound loci occurred only in the glucose-starved condition (Fig. 5a), consistent with data showing that Rst2 is predominantly active in glucose-deficient conditions [13]. Focusing on the glucose-starved condition, a significant overlap was observed between the protein-coding genes bound by Rst2 and Tup11, with 273 genes (64% of the total pool) bound by both factors (Fig. 5b). Furthermore, all but 14 of the 103 Scr1 bound genes from the glucose or sucrose conditions were bound by Rst2 and Tup11 in the glucose-starved condition (Fig. 5c), suggesting that Tup/Ssn6 acts with Scr1 or Rst2 at chromatin in a carbon-source dependent manner to enforce the repression or activation of CCR target genes.

The set of 273 Tup11 and Rst2 bound protein-coding genes was enriched for plasma membrane and cell wall components and a range of biological processes including hexose transport, hexose metabolism, glycolysis, gluconeogenesis, energy generation, ammonium transport, drug transport, ribosome biogenesis and RNA processing reflecting widespread regulatory roles of Rst2-Tup/Ssn6 in *S. pombe* cells under glucose starvation (Fig. 5d, Additional file 9: Table S8). The 50 protein-coding genes bound only by Rst2 were enriched for meiotic and conjugation biological processes reflecting the known roles of Rst2 in glucose-deficient conditions (Additional file 1: Figure S11B). In contrast, the 102 protein-coding genes bound only by Tup11 were enriched for iron assimilation, cellular iron homeostasis and oxidation-reduction processes (Additional file 1: Figure S11B) which is consistent with Tup11 localisation to iron metabolism genes through binding of the Fep1 transcription factor [44–46]. In line with their common gene targets, we observed similar global enrichment profiles of Tup11 and Rst2 in the glucose-starved condition (Fig. 5e) and co-localisation of both factors at the genes, such as *inv1*^+^ (Fig. 5f), which is upregulated in the absence of glucose [4]. These results suggest that Rst2 may bind the Tup/Ssn6 complex at chromatin in glucose-starved conditions and repurpose it for gene activation.

Consistent with this hypothesis, the *fbp1*^+^ and *ste11*^+^ genes, which are induced by Rst2 in the absence of glucose, were bound by both Rst2 and Tup11 [27, 40] (Additional file 9: Table S8). Examination of a complete set of 343 protein-coding and non-coding genes enriched for both Tup11 and Rst2 revealed widespread activation of Rst2 and Tup11 co-regulated genes in the glucose-starved condition. 198/343 (57.7%) of Tup11 and Rst2-bound loci showed increased expression levels suggesting that Tup/Ssn6 might contribute to the activation of these genes via association with Rst2 (Fig. 5g). Carbon metabolism and transcription factor encoding genes were enriched in this upregulated gene set, including the *ght* hexose transporters, the stress pathway transcriptional activators *atf1*^*+*^, *prr1*^*+*^, *rsv2*^*+*^*,* and *scr1*^*+*^ itself, implicating Rst2 and Tup11 in the activation of these factors. Together, these results suggest important roles for Rst2 and Tup11 in the activation of these genes in *S. pombe* in glucose-deficient conditions. Interestingly, several glycolysis, plasma membrane and cell wall genes were bound by Rst2 and Tup11 but repressed compared to glucose or sucrose conditions, suggesting that co-localisation of Rst2 with Tup11 is not sufficient for gene activation at all loci and that additional regulatory signals likely influence the regulation of these genes (Fig. 5g).

### Scr1, Tup11 and Rst2 co-localise at a subset of carbon metabolism genes in glucose-sufficient conditions

**Fig. 6:**
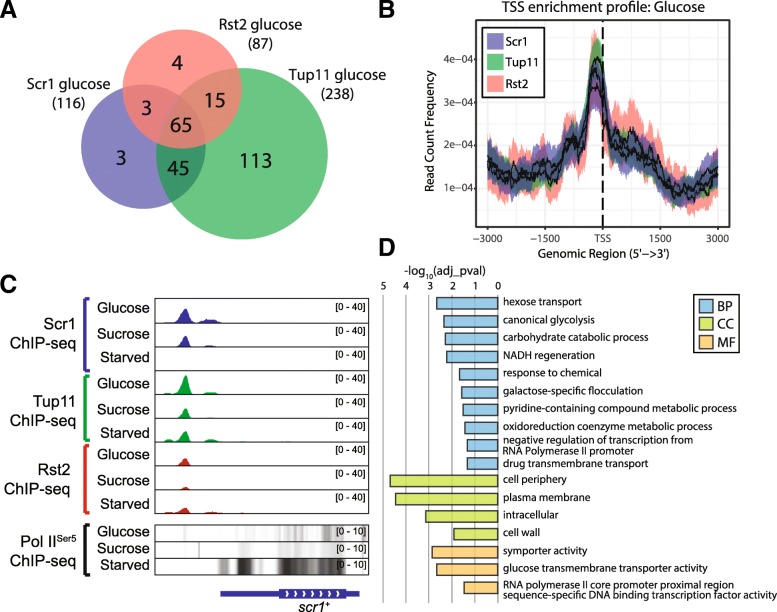
Scr1, Tup11 and Rst2 co localize at gene promoters in the presence of glucose. **a** Overlap of protein-coding genes bound by Scr1, Tup11 and Rst2 in glucose. **b** Enrichment profiles of Scr1, Tup11 and Rst2 surrounding *S. pombe* gene transcription start sites (TSS) in the glucose condition. Solid line indicates the mean while the colored region defines a 95% confidence interval. **c** Genome browser visualization of Scr1, Tup11, Rst2 and RNA PolII^Ser5^ enrichment at the *scr1*^+^ locus. The black arrows indicate significant regions of Scr1, Tup11 and Rst2 enrichment. **d** GO terms enriched in the subsets of protein-coding genes bound by Scr1, Rst2 and Tup11 in the glucose condition

In contrast to earlier work suggesting that Rst2 is predominantly excluded from the nucleus after phosphorylation by PKA in the presence of glucose [27], we observed significant Rst2 enrichment at 87 and 73 protein-coding genes in the glucose and sucrose conditions respectively (Additional file 8: Table S7). Furthermore, a subset of 51 protein-coding genes were bound by Rst2 in all three conditions, indicating that Rst2 remains bound to some loci regardless of the glucose concentration (Fig. 5a). We compared our ChIP-seq data for Rst2, Scr1 and Tup11 in the glucose condition, identifying 65 protein-coding genes bound by all three factors (Fig. 6a, Additional file 10: Table S9), and striking overlap in their global enrichment patterns in the glucose condition (Fig. 6b). Moreover, all three factors co-occupied the same genomic locations in the promoters of these genes. One key example of this was at the *scr1*^+^ promoter (Fig. 6c), suggesting that additional layers of regulation in concert with the autoregulatory capacity of Scr1 affect *scr1*^*+*^ expression. This set of 65 protein-coding genes bound by all three factors was enriched for hexose transport, glycolysis and transcription factor gene ontologies (Fig. 6d). These observations suggest that Scr1, Rst2 and Tup11 co-occupy and potentially co-operate to achieve transcriptional regulation of these genes in the presence of glucose.

**Fig. 7:**
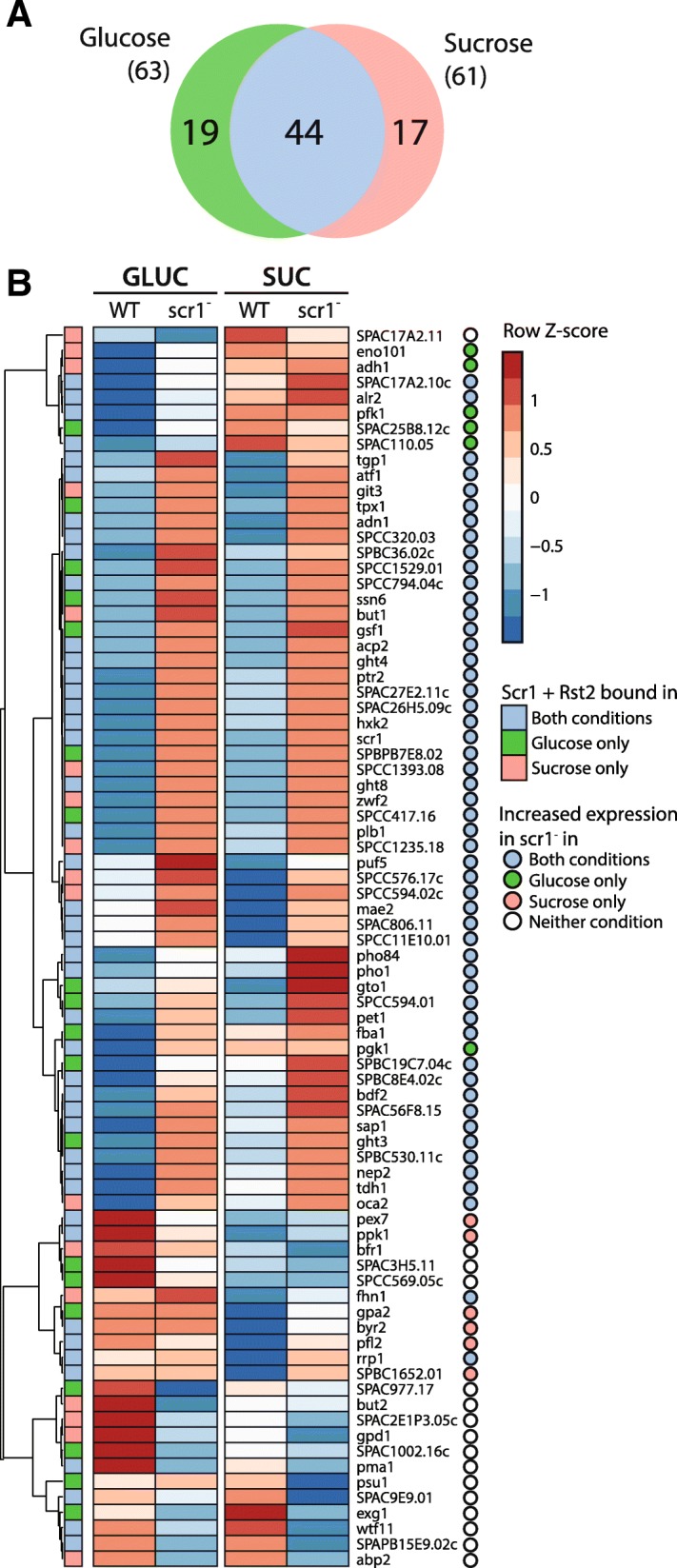
Genes bound by Scr1, Tup11 and Rst2 in glucose are predominantly upregulated in the *scr1*^−^ mutant. **a** Overlap of protein-coding genes bound by Scr1, Tup11 and Rst2 in glucose and sucrose conditions. **b** Heatmap of gene expression for each of the genes in **a** in wild type and *scr1*^−^ mutant cells in glucose or sucrose conditions. Coloured boxes indicate significant enrichment of both Scr1 and Rst2 at that gene in glucose (green), sucrose (red) or both conditions (blue). Coloured circles indicate increased expression in the *scr1*^−^ mutant background vs. wild type *S. pombe* for each gene in glucose (green), sucrose (red) or both conditions (blue). White circles indicate genes showing no increase in expression in the *scr1*^−^ mutant background relative to wild type *S. pombe* in either condition. The *fbp1*^+^ gene, while bound by Scr1 glucose and sucrose, is not shown due to a lack of significant enrichment of Rst2 in either condition

Since both Scr1 and Rst2 have been suggested to modulate Tup/Ssn6 function at the *fbp1*^+^ promoter [13], one possible explanation is that they compete for association with Tup/Ssn6 at these loci. Thus, the absence of one factor may “tip the balance” in favour of the opposing factor, which should result in changes to gene expression compared to wild type cells. To test this hypothesis, RNA-seq data from wild type and *scr1*^−^ mutant cells was examined for a non-redundant set of 80 protein-coding genes bound by Scr1, Tup11 and Rst2 in glucose and/or sucrose (Fig. 7a). This set included 13 of the 32 “Scr1-dependent genes” we defined earlier (Additional file 1: Figure S12). Consistent with our hypothesis that genes bound by both factors should increase in expression in the *scr1*^*−*^ mutant background, 52/80 (65%) genes displayed increased expression in the *scr1*^*−*^ mutant relative to wild type in both conditions, with a further 11 genes upregulated in either of the two conditions (Fig. 7b). Increased expression of a gene in the *scr1*^−^ mutant background was generally consistent with the presence of Scr1 and Rst2 co-localisation at that gene promoter. Overall, these data support the hypothesis that interplay between Scr1 and Rst2 at these loci in the presence of glucose may determine the role of Tup/Ssn6 in transcription. Since *S. pombe* lacks regulatory mechanisms found in other yeasts, such as the Snf3/Rgt2/Rgt1 system in *S. cerevisiae* [47], this feature may provide an avenue to rapidly modulate gene expression in the face of changing environmental carbon conditions.

## Discussion

This study sought to better understand the regulatory network of CCR genome-wide in *S. pombe*. Whereas previous examination of the CCR regulatory network in other fungi such as *S. cerevisiae* have relied on the identification of a consensus Mig1 DNA binding motif to assign whether genes are directly or indirectly regulated by CCR [18], we integrated RNA-seq and ChIP-seq approaches and defined a core set of 32 Scr1-dependent genes both bound by Scr1, and significantly upregulated in the *scr1*^−^ mutant background (Additional file 6: Table S5). The genes regulated by Scr1 operate across a range of canonical CCR related metabolic pathways and, interestingly, core stress responses and sexual differentiation, implicating Scr1 in the regulation of multiple biological processes in *S. pombe* (Figs. 1, 2 and 3).

**Fig. 8:**
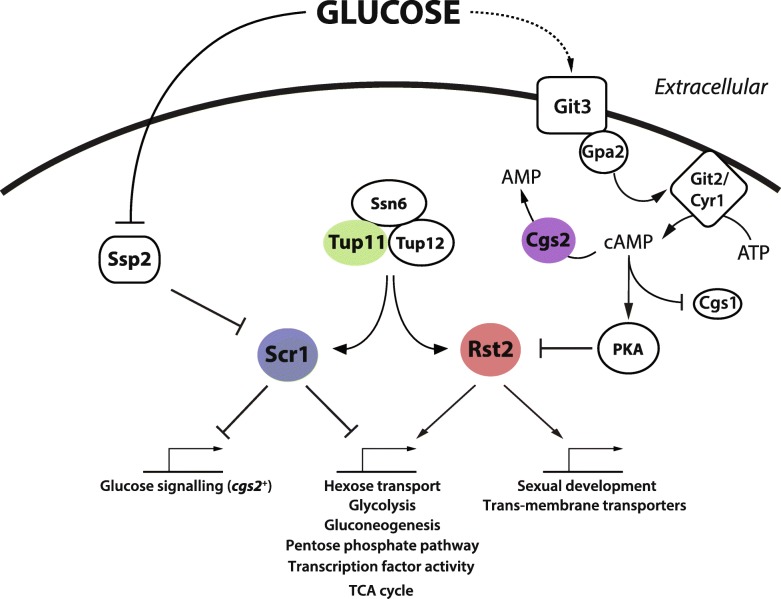
Scr1 and Rst2 co-function with Tup/Ssn6 to coordinate CCR in *S. pombe*. The Scr1 repressor (blue) and the Rst2 activator (red) comprise two downstream effectors of the glucose signal in *S. pombe*. In the glucose condition, nuclear localized Scr1 binds to and enforces the repression of genes involved in carbohydrate utilization, hexose transport, glucose signaling, glycolysis and the TCA cycle. Under these conditions the Git3-Gpa2-Git2 glucose sensing machinery is also active and sustains intracellular cAMP levels via Cyr1, in turn maintaining PKA in an active state via Cgs1 inhibition resulting in the hyper-phosphorylation and inactivation of Rst2. In the absence of glucose, Scr1 is inactivated by the Ssp2 AMPK [17], Cgs2 (purple) is subsequently upregulated and drives the conversion of intracellular cAMP to AMP [51]. This removes cAMP mediated inhibition of the PKA regulatory module Cgs1, allowing inhibition of PKA activity. In turn, Rst2 becomes active and replaces Scr1 at the promoter of the majority of Scr1 target genes as well as genes involved in gluconeogenesis and sexual differentiation. (Fig. 5c). Tup11 (green), a component of the Tup/Ssn6 co-regulator, co-localises at gene promoters with both Scr1 and Rst2 regardless of carbon condition thus playing a role in both the repression and activation of gene expression in response to glucose

Homothallic *scr1*Δ mutants exhibited ectopic meiosis in carbon and nitrogen replete conditions suggesting that Scr1 also acts to repress sexual differentiation (Fig. 3). While multiple meiosis genes, including *ste11*^+^ and *mei2*^+^, were indirectly regulated by Scr1, *cgs2*^+^ was both directly bound by Scr1 and significantly upregulated in the *scr1*^−^ mutant background in glucose-sufficient conditions (Fig. 3a). Cgs2 converts cellular cAMP to AMP, reducing cAMP levels within the cell and relieving cAMP-dependent inhibition of Cgs1, which is subsequently able to inhibit PKA [39, 48–51] (Fig. 3f). Thus, Cgs2 is an indirect inhibitor of PKA (Fig. 8). Inhibition of PKA results in the induction of a range of genes involved in gluconeogenesis, stress and meiosis including *fbp1*^+^ and *ste11*^+^ via Rst2, the ATF/CREB heterodimeric activator, Atf1-Pcr1, and the Spt-Ada-Gcn5-acetyltransferase (SAGA) transcriptional co-activator complex [40, 52, 53]. We have shown that *cgs2*^+^*,* is a direct target of Scr1 and is significantly upregulated under glucose-sufficient conditions in the absence of *scr1*^−^, thus linking the glucose-sensing cAMP-dependent PKA pathway with the Scr1-mediated CCR response. In previous work, overexpression of *cgs2*^+^ in *S. pombe* resulted in a decrease in cellular cAMP and ectopic meiosis in nutrient replete conditions [54] suggesting that ectopic expression of *cgs2*^+^ may be sufficient to drive activation of meiosis genes in the presence of glucose via misregulation of PKA signaling. If so, it would suggest that Scr1 indirectly contributes to the suppression of the *S. pombe* sexual differentiation pathway via transcriptional control of *cgs2*^+^ under favorable carbon conditions.

The enrichment of HAP complex and ATF/CREB factor binding motifs in the promoter of Scr1 regulated genes suggests that these factors also influence the regulation of CCR target genes in *S. pombe* (Additional file 1: Figure S7). The HAP complex, ATF/CREB (e.g. Atf1) and Rst2 also regulate key Scr1 target genes such as *fbp1*^+^ [24, 27]. Importantly, the *atf1*^+^ promoter was bound by Scr1 in glucose-sufficient conditions suggesting CCR acts to repress the transcriptional programs regulated by Atf1 when glucose is replete (Additional file 6: Table S5). Atf1 responds to Sty1 MAPK signaling to activate gene expression under various conditions including, UV, oxidative and osmotic stress [55–59], and regulates cAMP dependent PKA signaling via modulation of chromatin structure at the aforementioned *cgs2*^+^ locus [51]. Atf1 is also an activator of *ste11*^+^ expression, thereby linking mating to Sty1-mediated stress responses in *S. pombe* [55, 60]. Unlike *S. cerevisiae*, which readily mates in nutrient rich media and undergoes meiosis only under nutrient limitation [2], *S. pombe* mates only in conditions of nutrient or physiological stress and usually undergoes sporulation immediately following meiosis [61]. Interestingly, *atf1*^+^ was not significantly upregulated in the *scr1*^−^ mutant background, suggesting other factors may be required for full *atf1*^+^ induction, such as Rst2, which, along with Tup11, was bound at *atf1*^+^ in the glucose-starved condition, where *atf1*^+^ was upregulated (Additional file 8: Table S7). While it is logical that Scr1 should repress genes that promote meiosis when nutrients are replete, our data suggest that Scr1-mediated CCR may also repress the Sty1 mitogen-activated protein kinase (MAPK) signaling pathway in *S. pombe* via regulation of *atf1*^+^. Thus, Scr1 may additionally function as a repressor of the nutrient stress response in glucose-sufficient conditions.

In addition to Scr1, we defined the regulon of Tup11 and Rst2 in glucose, sucrose and glucose-starved conditions. We observed co-localisation of Scr1 and Tup11 at almost all Scr1 bound sites in glucose and sucrose conditions (Fig. 4b & c) and determined through co-immunoprecipitation that the two proteins physically interact (Additional file 1: Figure S10). Thus, like *S. cerevisiae*, targeting of the Tup/Ssn6 complex by the primary CCR transcription factor is conserved in *S. pombe* [16, 62]. Contrary to previous mechanistic models of CCR whereby Tup/Ssn6 complexes are recruited by CCR effectors to the DNA in the presence of glucose, and are dissociated from the DNA in the absence of glucose [63, 64], *S. cerevisiae* Tup1/Ssn6 remains at certain loci following glucose depletion, and in some conditions can act as a co-activator of gene expression, possibly by facilitating recruitment of the SWI/SNF, Mediator and/or SAGA complexes [65–68]. Consistent with these observations, just 30 protein-coding genes (7.4% of all Tup11 targets) associated with Tup11 enrichment were observed only in glucose or sucrose and not in glucose-starved conditions (Fig. 4a). This suggests that Tup11 remains bound to the majority of target loci regardless of the local glucose concentration or is displaced and subsequently re-recruited by other transcription factors within the timepoints analysed in this study. We found that Scr1 is replaced by Rst2 in glucose-starved conditions at CCR genes that also show Tup11 enrichment, increased expression and increased RNA Pol II^Ser5^ enrichment compared to glucose-sufficient conditions, supporting the hypothesis that Tup/Ssn6 function can be toggled between repressing and activating functions via association with different transcription factors (Fig. 8). Previous work suggests that the transcription factors Rst2, Atf1 and the CCAAT binding complex affect chromatin architecture to counteract the repressive capability of Tup/Ssn6 at *fbp1*^+^ [69, 70]. These studies, combined with data showing that loss of *tup11*^+^ and *tup12*^+^ is sufficient to derepress *fbp1*^+^ in the presence of glucose [24] argues against a direct role for Tup/Ssn6 in gene activation. In contrast, the *inv1*^+^ promoter element SPCC191.10, which is bound by Rst2 and Tup11 in glucose-starved conditions (Fig. 5f) was shown under the same conditions to also recruit the SWI/SNF chromatin remodelling complex and the SAGA histone acetyltransferase complex which are required for *inv1*^+^ induction [71]. This suggests that Tup/Ssn6 might be involved in the recruitment of SWI/SNF and SAGA at *inv1*^+^ and possibly other loci in glucose-starved conditions to promote gene activation in *S. pombe* in a similar fashion to *S. cerevisiae*. While the co-localisation of Rst2 and Tup11 binding at actively transcribed genes implies co-operation toward gene activation in *S. pombe*, the mechanism underlying this process remains unclear and may be different at distinct loci.

In contrast to previous work showing that Rst2 is predominantly excluded from the nucleus in the presence of glucose [27], we observed Rst2 enrichment at 87 protein-coding genes under these conditions confirming that Rst2 plays functional roles in both the presence and absence of glucose (Fig. 6a). Our data support a model for differential control, whereby a subset of genes are bound by Rst2 in the presence of glucose while a wider range of Rst2 targets are bound only in glucose-starved conditions (Fig. 7a, Additional file 8: Table S7). While it is unclear what determines the subset of Rst2 target genes that are bound by Rst2 in glucose compared to glucose-starved conditions, it has been shown that nitric oxide can induce Rst2 activity in the presence of glucose [72], suggesting that additional Rst2 control mechanisms exist which may regulate its localisation and/or function in glucose-sufficient conditions.

Rst2 replaces Scr1 at the *fbp1*^+^ promoter upon glucose starvation to induce gene expression [13]. Our data suggests that this pattern extends to most Scr1 bound genes which are reciprocally bound by Rst2 in the glucose-starved condition where Scr1 is absent (Fig. 5c). Given that Scr1 and Tup/Ssn6 co-localisation in the presence of glucose correlates with repression (Fig. 4e & f), while Rst2 and Tup/Ssn6 co-localisation in the absence of glucose correlates with activation (e.g. *fbp1*^+^ and *ste11*^*+*^, Fig. 5g) [13, 40], co-localisation of all three factors may indicate competition between Scr1 and Rst2 for association with the Tup/Ssn6 complex and control over gene expression. Scr1, Tup11 and Rst2 co-localised at 65 protein-coding genes functioning primarily in carbon metabolism pathways (Fig. 6a & d). Our RNA-seq data showed that genes bound by Scr1, Tup11 and Rst2 in the glucose or sucrose conditions mostly increased in expression in the *scr1*^−^ mutant background (Fig. 7b) supporting the idea that Scr1 and Rst2 compete for control over these loci, and that in the absence of Scr1, Rst2 can force their activation, possibly by modulating the function of Tup/Ssn6 or its ability to recruit downstream chromatin remodelling machinery. Competition between transcription factors has been reported in *S. cerevisiae*, where the Yox1 transcriptional repressor competes with the forkhead transcriptional activator, Fkh2, for binding to the Mcm1 protein at a subset of Mcm1 regulated cell-cycle genes [73]. A caveat to this model of Scr1 and Rst2 competition was that some genes displayed reduced expression in the *scr1*^−^ mutant background compared to wild type, suggesting additional regulatory factors are likely necessary for the control of these genes.

An alternative explanation for Scr1 and Rst2 co-localisation at these loci in glucose-sufficient conditions may be that the two proteins co-operate to provide additional regulatory complexity to the *S. pombe* cell. In *S. cerevisiae*, a subset of Mig1 target genes is also regulated by the glucose sensing pathway Snf3/Rgt2/Rgt1, and the Mig1 orthologues Mig2 and Mig3, in addition to the cAMP/PKA pathway [18, 74]. These pathways provide the budding yeast cell multiple layers of regulation to fine tune gene expression [2, 18, 74]. Given that *S. pombe* contains no Snf3/Rst2-like system, and the function of its closest Scr1 paralogue, Rsv1, appears specific to stationary phase [75, 76], co-occupancy of Scr1 (CCR) and Rst2 (cAMP/PKA) at CCR-regulated loci in the presence of glucose might maintain a “poised” transcriptional state, providing *S. pombe* an avenue to rapidly induce or repress transcription to optimise glucose uptake in response to fluctuations in glucose availability.

## Conclusions

We investigated the glucose-responsive transcriptional regulatory network of *S. pombe*, identifying striking differences in global transcription in wild type cells grown under distinct carbon conditions. Scr1, was required for the repression of genes involved in both canonical CCR pathways and nutrient stress induced sexual differentiation in glucose-sufficient conditions. Analysis of the genomic localization of Scr1, Tup11 and Rst2 suggested that the Tup/Ssn6 complex acts in concert with Scr1-mediated repression and Rst2-mediated activation on a genome wide scale. Finally, co-occupancy of all three factors at key carbon metabolism genes in the presence of glucose highlighted potential competition between the primary transcriptional repressor (Scr1) and activator (Rst2) of CCR for association with Tup/Ssn6 as a feature of the transcriptional regulatory network governing carbon metabolism in *S. pombe*.

## Methods

### *Schizosaccharomyces pombe* strains and growth conditions

No samples generated during this study were obtained from commercial sources. *S. pombe* strains used in this study are listed in Additional file 11: Table S10. Unless indicated, *S. pombe* was grown at 32 °C in yeast extract (YES) medium containing 3% (*w*/*v*) glucose with supplements [77]. For genetic crosses, parental strains were inoculated in equal proportions onto solid sporulation agar (SPAS) medium and cultured at 26 °C [77]. Diploid cells were isolated via intragenic complementation of *ade6*-m210 and *ade6*-m216 alleles followed by selection for adenine prototrophy on defined Edinburgh minimal medium (EMM) with 2% (w/v) glucose without adenine [77]. Tetrad dissection was performed using a Singer™ Sporeplay+ microscope. Microscopic images were acquired on an Olympus BX60 microscope with a SPOT CCD camera (Diagnostic Instruments).

### Strain construction

Oligonucleotides used in this study are listed in Additional file 12: Table S11. Linear DNA constructs for generating gene deletions or C-terminal TAP or FLAG tag fusion alleles were generated using a PCR-based allelic replacement approach [78]. To generate an *scr1*Δ strain, a construct containing the KanMX6 marker and 100 bp of *scr1*^+^ CDS flanking DNA at the 5′ and 3′ end was amplified from plasmid pFA6a [78] using oligonucleotides scr1-kan-KO-F and scr1-kan-KO-R. A freshly generated diploid *S. pombe* strain isolated from a cross of strains Y39 and Y50 was transformed with this construct via the lithium acetate method [77]. Diploid transformants containing an *scr1*Δ allele were selected on YES + 150 mg/mL G418 (“Geneticin”, Gibco), genotyped by colony PCR using oligonucleotides DV-03-F and DV-04-R, and sporulated resulting in the haploid *scr1*Δ strains D163 & D165 (Additional file 11: Table S10). Linear C-terminal TAP tagging constructs for *scr1*^+^, *tup11*^+^ and *rst2*^+^ were amplified from plasmid pFA6a-4X-CTAP [43] using oligonucleotides scr1-TAP-F and scr1-TAP-R, tup11-TAP-F and tup11-TAP-R and rst2-TAP-F and rst2-TAP-R respectively, transformed into Y50 and selected on YES + G418 as above. A homothallic (*h*^90^) *scr1*Δ strain was generated by crossing D163 (*h*^−^ *scr1*Δ) to the homothallic prototroph Y9 (*h*^90^). The mating type of *S. pombe* strains was determined by PCR using the oligonucleotides pombe-MAT1, pombe-MATp and pombe-MATm [79]. The percentage of zygotic asci was determined by counting the total number of cells in random fields of view up to a total cell number of approximately 1000 cells. In the same fields of view the number of zygotic asci were then counted. The percentage asci was calculated as the total number of asci multiplied by 2 (since each ascus arises from two cells) divided by the total count & multiplied by 100.

### RNA preparation and sequencing

Y44/Y39 (wild type), D59/D60 (*scr1*::*ura4*^*+*^), and D163/D165 (*scr1*Δ) cells were inoculated into pre-cultures containing 50 mL YES 3% (*w*/*v*) glucose to initial OD_595_ ≈ 0.08 and cultured for 4 h. Following this, cells were collected by centrifugation (21 °C, 3 min, 900rcf), washed twice with sterile water and split evenly across 50 mL YES 3% (w/v) glucose, YES 3% (w/v) sucrose, YES 3% (*v*/v) glycerol + 0.1% (w/v) glucose or YES 3% (v/v) glycerol cultures. Total RNA from two independent biological replicates for each strain and experimental condition was prepared using hot-phenol extraction [80]. RNA-seq library construction was performed in three separate batches by the Australian Genome Research Facility using Illumina TruSeq unstranded (batch 1) and TruSeq stranded (batches 2 and 3) protocols. Sequencing and quality metrics of RNA-seq and ChIP-seq libraries are shown in Additional file 13: Table S12 and Additional file 14: Table S13 respectively.

### Tandem affinity purification (TAP) & immunoblotting

TAP was performed on *S. pombe* strains Y50/Y39 (wild type), D51/D52 (Scr1-TAP), D96 (Tup11-TAP), D202/205 (Scr1-TAP + Tup11-FLAG) and D206/207 (Tup11-TAP + Scr1-FLAG) (Additional file 11: Table S10) as described previously [43]. To prepare whole cell lysates for TAP, cells were cultured in 4 L YES 3% (w/v) glucose to OD_595_ ≈ 0.5–0.7, harvested by centrifugation, transferred to stainless steel canisters, cryogenically frozen in liquid nitrogen, and lysed in a Retsch™ Mixer Mill MM200 using 5 × 2 minute cycles at maximum speed. Whole cell lysates were used as input for the TAP procedure. Immunoblotting of whole-cell and purified lysates from TAP and FLAG tagged strains was performed using either Rabbit anti-TAP (Thermo Fisher #CAB1001) primary or an in-house derived Mouse anti-FLAG-M2 primary, both at 1:1000 dilution. Anti-Rabbit/Mouse horseradish peroxidase (HRP) conjugated secondary antibodies were used at 1:10,000 dilution.

### Chromatin immunoprecipitation (ChIP) and ChIP-seq library preparation

ChIP was performed as described previously [81] using two independent biological replicates for each strain and experimental condition. To prepare chromatin for ChIPs, cells cultured in YES 3% (w/v) glucose to OD_595_ ≈ 0.5 were harvested by centrifugation, washed twice in sterile water, and split evenly across 50 mL YES 3% (w/v) glucose, YES 3% (w/v) sucrose or YES 3% (v/v) glycerol. After a further 4 h of culturing, cells were crosslinked with 1% (v/v) formaldehyde for 15 min at room temperature with mild shaking. Crosslinking was quenched with 2.5 mL 2.5 M glycine and cells were lysed by bead beating. The chromatin fraction was sonicated to 200-500 bp fragments using a Bioruptor sonicator (Diagenode) on high power using 5 × 9 min cycles of 30 s ON, 60 s OFF. For TAP-tag ChIPs, 4uL mouse anti-protein A antibody (Sigma P2921) was coupled to 100uL pan-mouse Dynabeads (Invitrogen). RNA PolII^Ser5^ ChIPs were conducted in either wild type (Y9) or Scr1-TAP (D51/D52) backgrounds. An excess (5uL) of mouse anti-RNA Polymerase II^Ser5^ phospho-CTD antibody (clone 3E8, Merck 04–1572) was coupled to 100uL goat anti-mouse Dynabeads (Invitrogen) to saturate the beads with antibody and prevent cross-reactivity with the Protein A moiety of the TAP tag. ChIP-seq DNA was quantified using a high-sensitivity DNA assay on a Qubit 2.0 fluorometer (Invitrogen). ChIP-seq library preparation was performed as described previously [82]. Library DNA was examined for quality on an Agilent Bioanalyser 2100 using DNA high-sensitivity chips. ChIP-seq DNA was sequenced on an Illumina HiSeq 2500 using either a 75 bp single-end or 100 bp paired-end strategy. Sequencing and quality metrics of ChIP-seq libraries are shown in Additional file 14: Table S13.

### RNA-seq and ChIP-seq data analysis

Illumina sequencing reads were adapter and quality trimmed using Cutadapt (v1.9.1) [83] with default parameters and assessed for quality using FastQC (v0.11.5) (http://www.bioinformatics.babraham.ac.uk/projects/fastqc/). Read data was aligned to the *S. pombe* reference genome (ASM294v2.25) using Bowtie2 v2.3.1 (ChIP-seq samples) [84] or Tophat2 v2.1.1 (RNA-seq samples) [85]. Bowtie2 parameters were set as: --very-sensitive-local; −-minins 0; −-maxins 600; −-threads 8. Tophat2 parameters were as follows: --max-intron-size 5000 --library-type fr-firststrand (for stranded samples), fr-unstranded (for unstranded samples). Reads mapping to features were counted with featureCounts v1.5.2 [86] using the following parameters: -O -M -T 4. For stranded libraries, the -s 2 parameter was used. Differential gene expression analysis was performed using DESeq2 (v1.12.3) accounting for sequencing batch in the experimental design [87]. Genes with a log_2_ fold change (Log_2_FC) greater than 1 or less than − 1 and a False Discovery Rate (FDR) adjusted *p*-value (P_adj_) less than 0.05 were considered differentially expressed and used for further analysis. ChIP-seq peak detection was performed using a workflow based on MACS v2.1.1 [88] coupled with irreproducible discovery rate (IDR) correction across replicates as described previously [89]. An input sample was used to provide background enrichment correction for each carbon condition. Deeptools2 v2.5.3 [90] and ChIPseeker [91] were used for visualization of input normalized ChIP-seq samples. Gene ontology and gene set enrichment analyses were performed using AnGeLi with default parameters [92].

## Additional files

Additional file 1: **Figure S1.** Fungal CCR effectors are conserved within the zinc finger DNA binding domain. **Figure S2.** Glucose availability directly influences the transcriptional program of *S. pombe.* **Figure S3.** Primary carbon metabolism pathways are upregulated in the absence of glucose. **Figure S4.** *scr1*::*ura4*^+^ disruption is a faithful representation of *scr1*^−^ loss of function. **Figure S5.** Generation of a TAP-epitope tagged Scr1 expression *S. pombe* strain. **Figure S6.** Scr1 possesses autoregulatory capacity. **Figure S7.** The promoters of Scr1-dependent genes contain putative Scr1, HAP complex and ATF/CREB factor binding sites. **Figure S8.** *scr1*Δ *h*^90^ cells mate normally on SPAS medium at 26 degrees. **Figure S9.** Scr1 and Tup11 co-localise at the promoter of known Scr1 target genes. **Figure S10.** Scr1 and the Tup/Ssn6 complex physically interact but do not form a stable stoichiometric protein complex. **Figure S11.** GO Enrichment of Rst2 and Tup11 independent gene targets. **Figure S12.** Overlap of protein coding genes bound by Scr1, Tup11 and Rst2 in glucose or sucrose and the “Scr1-dependent” gene set. (DOCX 8239 kb)

Additional file 2: **Table S1**. Results of RNA-seq analysis for protein-coding genes in wild type cells cultured in glucose, sucrose, glucose-deficient or glucose-starved conditions. (XLSX 1356 kb)

Additional file 3: **Table S2.** Results of RNA-seq analysis for non-coding RNAs or other small RNAs in wild type cells cultured in glucose, sucrose, glucose-deficient or glucose-starved conditions. (XLSX 450 kb)

Additional file 4: **Table S3.** Results of RNA-seq analysis for scr1 mutant cells vs wild type cells cultured in glucose or sucrose conditions. (XLSX 1801 kb)

Additional file 5: **Table S4.** Scr1-regulated genes under glucose sufficient conditions. (XLSX 17 kb)

Additional file 6: **Table S5.** Results of Scr1 ChIP-seq analysis. (XLSX 44 kb)

Additional file 7: **Table S6.** Results of Tup11 ChIP-seq analysis. (XLSX 69 kb)

Additional file 8: **Table S7.** Results of Rst2 ChIP-seq analysis. (XLSX 50 kb)

Additional file 9: **Table S8.** Analysis of shared and unique Tup11 and Rst2 ChIP-seq targets in glucose, sucrose and glucose-starved conditions. (XLSX 126 kb)

Additional file 10: **Table S9.** Analysis of shared and unique Scr1, Tup11 and Rst2 ChIP-seq targets. (XLSX 29 kb)

Additional file 11: **Table S10.** Strains used in this study. (XLSX 10 kb)

Additional file 12: **Table S11.** Oligos used in this study. (XLSX 10 kb)

Additional file 13: **Table S12.** List of RNA-seq samples. (XLSX 10 kb)

Additional file 14: **Table S13.** List of ChIP-seq samples. (XLSX 11 kb)

## Abbreviations

AMPK: AMP-activated protein kinase
CCR: Carbon catabolite repression
ChIP-seq: Chromatin immunoprecipitation sequencing
DEG: Differentially expressed gene
EMM: Edinburgh minimal medium
GO: Gene ontology
HRP: Horseradish peroxidase
MAPK: Mitogen-activated protein kinase
PKA: Protein kinase A
RNA-seq: RNA sequencing
SAGA: Spt-Ada-Gcn5-acetyltransferase complex
SPAS: Sporulation agar plus supplements
TAP: Tandem affinity purification
TCA cycle: Tricarboxylic citric-acid cycle
TSS: Transcription start sites
UV: Ultraviolet
YES: Yeast extract plus supplements medium

## References

[1] Bahn YS, Xue C, Idnurm A, Rutherford JC, Heitman J, Cardenas ME (2007). Sensing the environment: lessons from fungi. Nat Rev Microbiol.

[2] Broach JR (2012). Nutritional control of growth and development in yeast. Genetics.

[3] Diaz-Ruiz R, Rigoulet M, Devin A (2011). The Warburg and Crabtree effects: on the origin of cancer cell energy metabolism and of yeast glucose repression. Biochim Biophys Acta.

[4] Tanaka N, Ohuchi N, Mukai Y, Osaka Y, Ohtani Y, Tabuchi M, Bhuiyan MS, Fukui H, Harashima S, Takegawa K (1998). Isolation and characterization of an invertase and its repressor genes from *Schizosaccharomyces pombe*. Biochem Biophys Res Commun.

[5] Lundin M, Nehlin JO, Ronne H (1994). Importance of a flanking AT-rich region in target site recognition by the GC box-binding zinc finger protein MIG1. Mol Cell Biol.

[6] Cubero B, Scazzocchio C (1994). Two different, adjacent and divergent zinc finger binding sites are necessary for CREA-mediated carbon catabolite repression in the proline gene cluster of *Aspergillus nidulans*. EMBO J.

[7] Tzamarias D, Struhl K (1994). Functional dissection of the yeast Cyc8-Tup1 transcriptional co-repressor complex. Nature.

[8] Ca K, Redd MJ, Schultz J, Carlson M, Johnson AD (1992). Ssn6-Tup1 is a general repressor of transcription in yeast. Cell.

[9] Smith RL, Johnson AD (2000). Turning genes off by Ssn6-Tup1: a conserved system of transcriptional repression in eukaryotes. Trends Biochem Sci.

[10] Malave TM, Dent SY (2006). Transcriptional repression by Tup1-Ssn6. Biochem Cell Biol.

[11] Wong KH, Struhl K (2011). The Cyc8-Tup1 complex inhibits transcription primarily by masking the activation domain of the recruiting protein. Genes Dev.

[12] Rizzo JM, Mieczkowski PA, Buck MJ (2011). Tup1 stabilizes promoter nucleosome positioning and occupancy at transcriptionally plastic genes. Nucleic Acids Res.

[13] Hirota K, Hoffman CS, Ohta K (2006). Reciprocal nuclear shuttling of two antagonizing Zn finger proteins modulates Tup family corepressor function to repress chromatin remodeling. Eukaryot Cell.

[14] De Vit MJ, Waddle JA, Johnston M (1997). Regulated nuclear translocation of the Mig1 glucose repressor. Mol Biol Cell.

[15] Strauss J, Horvath HK, Abdallah BM, Kindermann J, Mach RL, Kubicek CP (1999). The function of CreA, the carbon catabolite repressor of Aspergillus nidulans, is regulated at the transcriptional and post-transcriptional level. Mol Microbiol.

[16] Papamichos-Chronakis M, Gligoris T, Tzamarias D (2004). The Snf1 kinase controls glucose repression in yeast by modulating interactions between the Mig1 repressor and the Cyc8-Tup1 co-repressor. EMBO Rep.

[17] Matsuzawa T, Fujita Y, Tohda H, Takegawa K (2012). Snf1-like protein kinase Ssp2 regulates glucose derepression in *Schizosaccharomyces pombe*. Eukaryot Cell.

[18] Westholm JO, Nordberg N, Muren E, Ameur A, Komorowski J, Ronne H (2008). Combinatorial control of gene expression by the three yeast repressors Mig1, Mig2 and Mig3. BMC Genomics.

[19] Hu Z, Killion PJ, Iyer VR (2007). Genetic reconstruction of a functional transcriptional regulatory network. Nat Genet.

[20] Mogensen J, Nielsen HB, Hofmann G, Nielsen J (2006). Transcription analysis using high-density micro-arrays of *Aspergillus nidulans* wild type and *creA* mutant during growth on glucose or ethanol. Fungal Genet Biol.

[21] Antonieto AC, dos Santos CL, Silva-Rocha R, Persinoti GF, Silva RN (2014). Defining the genome-wide role of CRE1 during carbon catabolite repression in *Trichoderma reesei* using RNA-Seq analysis. Fungal Genet Biol.

[22] Antonieto AC, de Paula RG, Castro Ldos S, Silva-Rocha R, Persinoti GF, Silva RN (2016). Trichoderma reesei CRE1-mediated carbon catabolite repression in re-sponse to Sophorose through RNA sequencing analysis. Curr Genomics.

[23] Matsuzawa T, Ohashi T, Hosomi A, Tanaka N, Tohda H, Takegawa K (2010). The *gld1*^+^ gene encoding glycerol dehydrogenase is required for glycerol metabolism in *Schizosaccharomyces pombe*. Appl Microbiol Biotechnol.

[24] Janoo RT, Neely LA, Braun BR, Whitehall SK, Hoffman CS (2001). Transcriptional regulators of the *Schizosaccharomyces pombe* fbp1 gene include two redundant Tup1p-like corepressors and the CCAAT binding factor activation complex. Genetics.

[25] Saitoh S, Mori A, Uehara L, Masuda F, Soejima S, Yanagida M (2015). Mechanisms of expression and translocation of major fission yeast glucose transporters regulated by CaMKK/phosphatases, nuclear shuttling, and TOR. Mol Biol Cell.

[26] Mukai Y, Matsuo E, Roth SY, Harashima S (1999). Conservation of histone binding and transcriptional repressor functions in a *Schizosaccharomyces pombe* Tup1p homolog. Mol Cell Biol.

[27] Higuchi T, Watanabe Y, Yamamoto M (2002). Protein kinase a regulates sexual development and gluconeogenesis through phosphorylation of the Zn finger transcriptional activator Rst2p in fission yeast. Mol Cell Biol.

[28] Steensels J, Snoek T, Meersman E, Picca Nicolino M, Voordeckers K, Verstrepen KJ (2014). Improving industrial yeast strains: exploiting natural and artificial diversity. FEMS Microbiol Rev.

[29] Petrovic U (2015). Next-generation biofuels: a new challenge for yeast. Yeast.

[30] Nielsen J (2013). Production of biopharmaceutical proteins by yeast: advances through metabolic engineering. Bioengineered.

[31] DeRisi JL, Iyer VR, Brown PO (1997). Exploring the metabolic and genetic control of gene expression on a genomic scale. Science.

[32] Zaman S, Lippman SI, Schneper L, Slonim N, Broach JR (2009). Glucose regulates transcription in yeast through a network of signaling pathways. Mol Syst Biol.

[33] Brauer MJ, Saldanha AJ, Dolinski K, Botstein D (2005). Homeostatic adjustment and metabolic remodeling in glucose-limited yeast cultures. Mol Biol Cell.

[34] Malecki M, Bitton DA, Rodriguez-Lopez M, Rallis C, Calavia NG, Smith GC, Bahler J (2016). Functional and regulatory profiling of energy metabolism in fission yeast. Genome Biol.

[35] Spirek M, Benko Z, Carnecka M, Rumpf C, Cipak L, Batova M, Marova I, Nam M, Kim DU, Park HO (2010). *S. pombe* genome deletion project: an update. Cell Cycle.

[36] Kim DU, Hayles J, Kim D, Wood V, Park HO, Won M, Yoo HS, Duhig T, Nam M, Palmer G (2010). Analysis of a genome-wide set of gene deletions in the fission yeast *Schizosaccharomyces pombe*. Nat Biotechnol.

[37] Machanick P, Bailey TL (2011). MEME-ChIP: motif analysis of large DNA datasets. Bioinformatics.

[38] Ma W, Noble WS, Bailey TL (2014). Motif-based analysis of large nucleotide data sets using MEME-ChIP. Nat Protoc.

[39] Matviw H, Li J, Young D (1993). The *Schizosaccharomyces pombe pde1/cgs2* gene encodes a cyclic AMP phosphodiesterase. Biochem Biophys Res Commun.

[40] Kunitomo H, Higuchi T, Iino Y, Yamamoto M (2000). A zinc-finger protein, Rst2p, regulates transcription of the fission yeast *ste11*^+^ gene, which encodes a pivotal transcription factor for sexual development. Mol Biol Cell.

[41] Fagerstrom-Billai F, Wright AP (2005). Functional comparison of the Tup11 and Tup12 transcriptional corepressors in fission yeast. Mol Cell Biol.

[42] Fagerstrom-Billai F, Durand-Dubief M, Ekwall K, Wright AP (2007). Individual subunits of the Ssn6-Tup11/12 corepressor are selectively required for repression of different target genes. Mol Cell Biol.

[43] Gould KL, Ren L, Feoktistova AS, Jennings JL, Link AJ (2004). Tandem affinity purification and identification of protein complex components. Methods.

[44] Pelletier B, Beaudoin J, Mukai Y, Labbe S (2002). Fep1, an iron sensor regulating iron transporter gene expression in *Schizosaccharomyces pombe*. J Biol Chem.

[45] Pelletier B, Beaudoin J, Philpott CC, Labbe S (2003). Fep1 represses expression of the fission yeast Schizosaccharomyces pombe siderophore-iron transport system. Nucleic Acids Res.

[46] Znaidi S, Pelletier B, Mukai Y, Labbe S (2004). The Schizosaccharomyces pombe corepressor Tup11 interacts with the iron-responsive transcription factor Fep1. J Biol Chem.

[47] Johnston M, Kim JH (2005). Glucose as a hormone: receptor-mediated glucose sensing in the yeast *Saccharomyces cerevisiae*. Biochem Soc Trans.

[48] Gupta DR, Paul SK, Oowatari Y, Matsuo Y, Kawamukai M (2011). Multistep regulation of protein kinase a in its localization, phosphorylation and binding with a regulatory subunit in fission yeast. Curr Genet.

[49] Gupta DR, Paul SK, Oowatari Y, Matsuo Y, Kawamukai M (2011). Complex formation, phosphorylation, and localization of protein kinase a of Schizosaccharomyces pombe upon glucose starvation. Biosci Biotechnol Biochem.

[50] Matsuo Y, McInnis B, Marcus S (2008). Regulation of the subcellular localization of cyclic AMP-dependent protein kinase in response to physiological stresses and sexual differentiation in the fission yeast *Schizosaccharomyces pombe*. Eukaryot Cell.

[51] Davidson MK, Shandilya HK, Hirota K, Ohta K, Wahls WP (2004). Atf1-Pcr1-M26 complex links stress-activated MAPK and cAMP-dependent protein kinase pathways via chromatin remodeling of *cgs2*^*+*^. J Biol Chem.

[52] Helmlinger D, Marguerat S, Villen J, Gygi SP, Bahler J, Winston F (2008). The *S. pombe* SAGA complex controls the switch from proliferation to sexual differentiation through the opposing roles of its subunits Gcn5 and Spt8. Genes Dev.

[53] La N, Hoffman CS (2000). Protein kinase a and mitogen-activated protein kinase pathways antagonistically regulate fission yeast fbp1 transcription by employing different modes of action at two upstream activation sites. Mol Cell Biol.

[54] Mochizuki N, Yamamoto M (1992). Reduction in the intracellular cAMP level triggers initiation of sexual development in fission yeast. Mol Gen Genet.

[55] Takeda T, Toda T, Kominami K, Kohnosu A, Yanagida M, Jones N (1995). *Schizosaccharomyces pombe atf1*^+^ encodes a transcription factor required for sexual development and entry into stationary phase. EMBO J.

[56] Shiozaki K, Russell P (1996). Conjugation, meiosis, and the osmotic stress response are regulated by Spc1 kinase through Atf1 transcription factor in fission yeast. Genes Dev.

[57] Wilkinson MG, Samuels M, Takeda T, Toone WM, Shieh JC, Toda T, Millar JB, Jones N (1996). The Atf1 transcription factor is a target for the Sty1 stress-activated MAP kinase pathway in fission yeast. Genes Dev.

[58] Degols G, Russell P (1997). Discrete roles of the Spc1 kinase and the Atf1 transcription factor in the UV response of *Schizosaccharomyces pombe*. Mol Cell Biol.

[59] Chen D, Toone WM, Mata J, Lyne R, Burns G, Kivinen K, Brazma A, Jones N, Bahler J (2003). Global transcriptional responses of fission yeast to environmental stress. Mol Biol Cell.

[60] Ohmiya R, Yamada H, Kato C, Aiba H, Mizuno T (2000). The Prr1 response regulator is essential for transcription of *ste11*^+^ and for sexual development in fission yeast. Mol Gen Genet.

[61] Hoffman CS, Wood V, Fantes PA (2015). An ancient yeast for Young geneticists: a primer on the *Schizosaccharomyces pombe* model system. Genetics.

[62] Ma T, Carlson M (1995). Repression by SSN6-TUP1 is directed by MIG1, a repressor/activator protein. Proc Natl Acad Sci U S A.

[63] Gancedo JM (2008). The early steps of glucose signalling in yeast. FEMS Microbiol Rev.

[64] Gancedo JM (1998). Yeast carbon catabolite repression. Microbiol Mol Biol Rev.

[65] Conlan RS, Gounalaki N, Hatzis P, Tzamarias D (1999). The Tup1-Cyc8 protein complex can shift from a transcriptional co-repressor to a transcriptional co-activator. J Biol Chem.

[66] Papamichos-Chronakis M, Petrakis T, Ktistaki E, Topalidou I, Tzamarias D (2002). Cti6, a PHD domain protein, bridges the Cyc8-Tup1 corepressor and the SAGA coactivator to overcome repression at *GAL1*. Mol Cell.

[67] Proft M, Struhl K (2002). Hog1 kinase converts the Sko1-Cyc8-Tup1 repressor complex into an activator that recruits SAGA and SWI/SNF in response to osmotic stress. Mol Cell.

[68] Tanaka N, Mukai Y (2015). Yeast Cyc8p and Tup1p proteins function as coactivators for transcription of Stp1/2p-dependent amino acid transporter genes. Biochem Biophys Res Commun.

[69] Asada R, Takemata N, Hoffman CS, Ohta K, Hirota K (2015). Antagonistic controls of chromatin and mRNA start site selection by Tup family corepressors and the CCAAT-binding factor. Mol Cell Biol.

[70] Asada R, Umeda M, Adachi A, Senmatsu S, Abe T, Iwasaki H, Ohta K, Hoffman CS, Hirota K (2017). Recruitment and delivery of the fission yeast Rst2 transcription factor via a local genome structure counteracts repression by Tup1-family corepressors. Nucleic Acids Res.

[71] Ahn S, Spatt D, Winston F (2012). The *Schizosaccharomyces pombe inv1*^+^ regulatory region is unusually large and contains redundant cis-acting elements that function in a SAGA-and Swi/Snf-dependent fashion. Eukaryot Cell.

[72] Kato T, Zhou X, Ma Y (2013). Possible involvement of nitric oxide and reactive oxygen species in glucose deprivation-induced activation of transcription factor rst2. PLoS One.

[73] Darieva Z, Clancy A, Bulmer R, Williams E, Pic-Taylor A, Morgan BA, Sharrocks AD (2010). A competitive transcription factor binding mechanism determines the timing of late cell cycle-dependent gene expression. Mol Cell.

[74] Ozcan S, Johnston M (1999). Function and regulation of yeast hexose transporters. Microbiol Mol Biol Rev.

[75] Hao Z, Furunobu A, Nagata A, Okayama H (1997). A zinc finger protein required for stationary phase viability in fission yeast. J Cell Sci.

[76] Mata J, Wilbrey A, Bahler J (2007). Transcriptional regulatory network for sexual differentiation in fission yeast. Genome Biol.

[77] Sabatinos SA, Forsburg SL (2010). Molecular genetics of *Schizosaccharomyces pombe*. Methods Enzymol.

[78] Bahler J, Wu JQ, Longtine MS, Shah NG, McKenzie A, Steever AB, Wach A, Philippsen P, Pringle JR (1998). Heterologous modules for efficient and versatile PCR-based gene targeting in *Schizosaccharomyces pombe*. Yeast.

[79] Looke M, Kristjuhan K, Kristjuhan A (2011). Extraction of genomic DNA from yeasts for PCR-based applications. BioTechniques.

[80] Lyne R, Burns G, Mata J, Penkett CJ, Rustici G, Chen D, Langford C, Vetrie D, Bahler J (2003). Whole-genome microarrays of fission yeast: characteristics, accuracy, reproducibility, and processing of array data. BMC Genomics.

[81] Monahan BJ, Villen J, Marguerat S, Bahler J, Gygi SP, Winston F (2008). Fission yeast SWI/SNF and RSC complexes show compositional and functional differences from budding yeast. Nat Struct Mol Biol.

[82] Wong KH, Jin Y, Moqtaderi Z. Multiplex Illumina sequencing using DNA barcoding. Curr Protoc Mol Biol. 2013, Chapter 7:Unit 7 11. 10.1002/0471142727.mb0711s101. 10.1002/0471142727.mb0711s101 23288465

[83] Martin M. Cutadapt removes adapter sequences from high-throughput sequencing reads. EMBnet J. 2011;17(1):10-12. 10.14806/ej.17.1.200.

[84] Langmead B, Salzberg SL (2012). Fast gapped-read alignment with bowtie 2. Nat Methods.

[85] Kim D, Pertea G, Trapnell C, Pimentel H, Kelley R, Salzberg SL (2013). TopHat2: accurate alignment of transcriptomes in the presence of insertions, deletions and gene fusions. Genome Biol.

[86] Liao Y, Smyth GK, Shi W (2014). featureCounts: an efficient general purpose program for assigning sequence reads to genomic features. Bioinformatics.

[87] Love MI, Huber W, Anders S (2014). Moderated estimation of fold change and dispersion for RNA-seq data with DESeq2. Genome Biol.

[88] Zhang Y, Liu T, Meyer CA, Eeckhoute J, Johnson DS, Bernstein BE, Nusbaum C, Myers RM, Brown M, Li W (2008). Model-based analysis of ChIP-Seq (MACS). Genome Biol.

[89] Landt SG, Marinov GK, Kundaje A, Kheradpour P, Pauli F, Batzoglou S, Bernstein BE, Bickel P, Brown JB, Cayting P (2012). ChIP-seq guidelines and practices of the ENCODE and modENCODE consortia. Genome Res.

[90] Ramirez F, Ryan DP, Gruning B, Bhardwaj V, Kilpert F, Richter AS, Heyne S, Dundar F, Manke T (2016). deepTools2: a next generation web server for deep-sequencing data analysis. Nucleic Acids Res.

[91] Yu G, Wang LG, He QY (2015). ChIPseeker: an R/Bioconductor package for ChIP peak annotation, comparison and visualization. Bioinformatics.

[92] Bitton DA, Schubert F, Dey S, Okoniewski M, Smith GC, Khadayate S, Pancaldi V, Wood V, Bahler J (2015). AnGeLi: a tool for the analysis of gene lists from fission yeast. Front Genet.

