# Supplementary material for: A genome-wide analysis of carbon catabolite repression in Schizosaccharomyces pombe
Source: BMC Genomics. 2019 Mar 29;20:251. doi: 10.1186/s12864-019-5602-8 (PMC6440086; doi:10.1186/s12864-019-5602-8)
Supplement: Supplementary file 1 — Figure S1. Fungal CCR effectors are conserved within the zinc finger DNA binding domain. Figure S2. Glucose availability directly influences the transcriptional program of S. pombe. Figure S3. Primary carbon metabolism pathways are upregulated in the absence of glucose. Figure S4. scr1::ura4+ disruption is a faithful representation of scr1− loss of function. Figure S5. Generation of a TAP-epitope tagged Scr1 expression S. pombe strain. Figure S6. Scr1 possesses autoregulatory capacity. Figure S7. The promoters of Scr1-dependent genes contain putative Scr1, HAP complex and ATF/CREB factor binding sites. Figure S8. scr1Δ h90 cells mate normally on SPAS medium at 26 degrees. Figure S9. Scr1 and Tup11 co-localise at the promoter of known Scr1 target genes. Figure S10. Scr1 and the Tup/Ssn6 complex physically interact but do not form a stable stoichiometric protein complex. Figure S11. GO Enrichment of Rst2 and Tup11 independent gene targets. Figure S12. Overlap of protein coding genes bound by Scr1, Tup11 and Rst2 in glucose or sucrose and the “Scr1-dependent” gene set. (DOCX 8239 kb) [file 12864_2019_5602_MOESM1_ESM.docx]

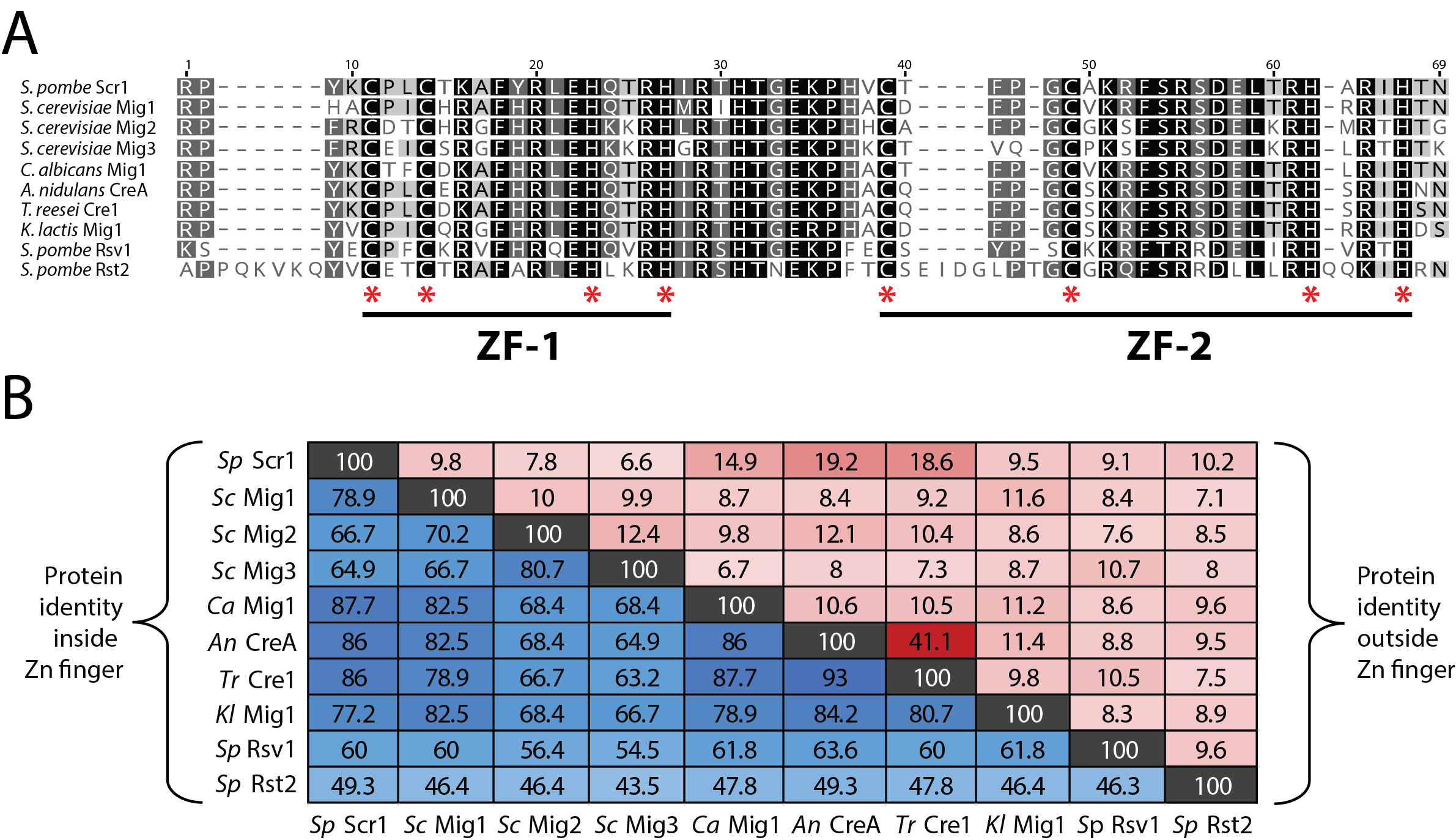


**Figure S1. Fungal CCR effectors are conserved within the zinc finger DNA binding domain.** A) Amino acid sequence alignment of the twin C_2_H_2_ zinc finger DNA-binding domains of *S. pombe* Scr1, *S. cerevisiae* Mig1, Mig2 and Mig3, *Candida albicans* Mig1, *A. nidulans* CreA, *T. reesei* Cre1 and *Kluyveromyces lactis* Mig1. Two additional C_2_H_2_ zinc finger domain containing proteins from *S. pombe*, Rst2 and Rsv1, are also shown. Asterisks indicate the positions of the conserved cysteine and histidine residues in the first (ZF-1) and second (ZF-2) zinc finger regions. B) Amino acid sequence identity contingency table of the proteins shown in (A). Numbers represent percentage protein sequence identity for each pairwise comparison. Blue shaded area indicates identity within the zinc finger domain (shown in A). Red shaded area indicates identity outside the zinc finger region. Intensity of coloring correlates with the degree of conservation. *Sp* = *Schizosaccharomyces pombe*, *Sc* = *Saccharomyces cerevisiae*, *Ca* = *Candida albicans*, *Tr* = *Trichoderma reesei*, *An* = *Aspergillus nidulans*, *Kl* = *Kluyveromyces lactis*.


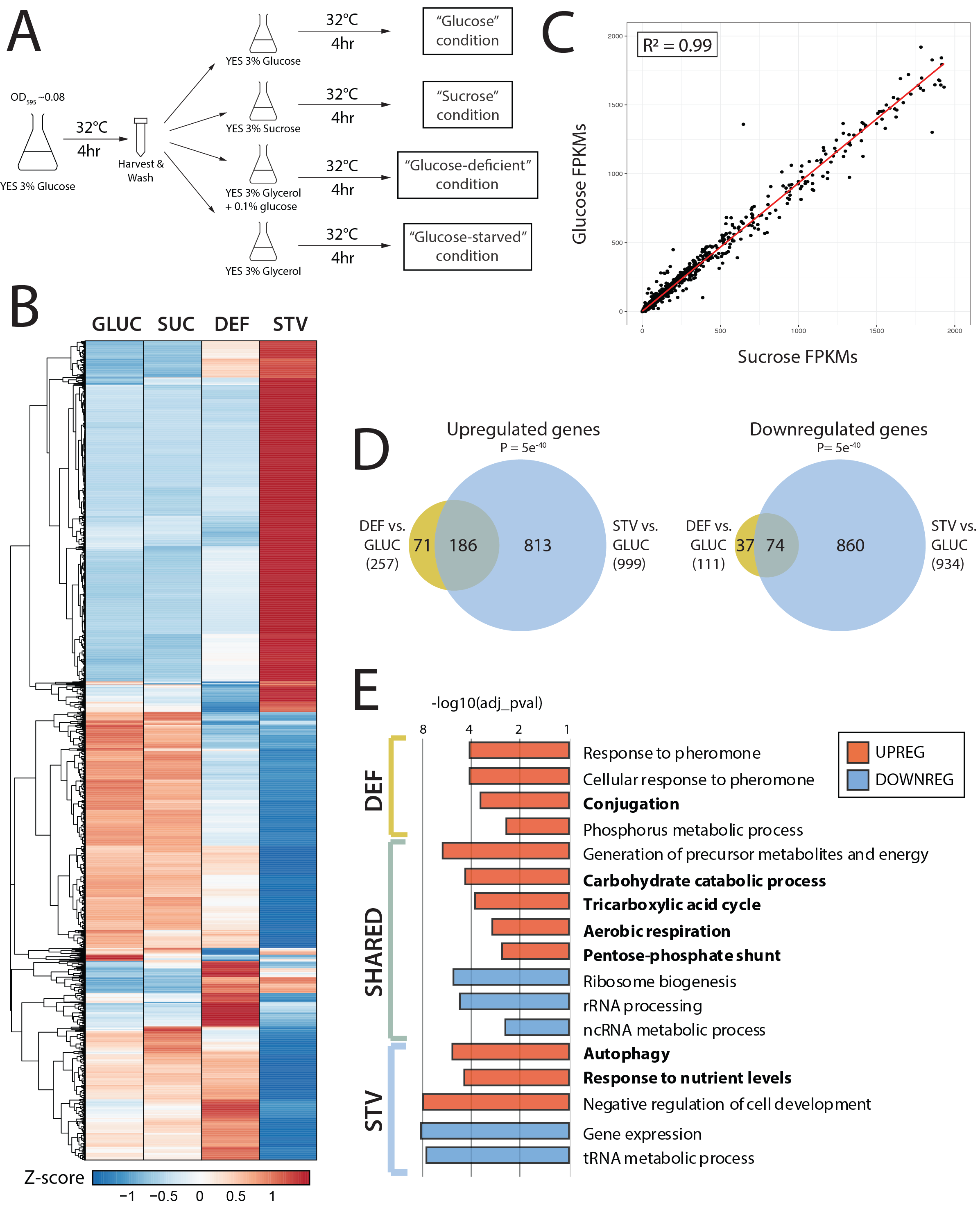


**Figure S2. Glucose availability directly influences the transcriptional program of *S. pombe.*** A) Schematic of shift culture experiments used in this study. *S. pombe* was cultured for four hours in YES 3% glucose before being harvested, washed and split across four different conditions, YES 3% glucose, YES 3% sucrose, YES 3% glycerol + 0.1% glucose or YES 3% glycerol and cultured for a further four hours at which point cells were harvested for RNA-extraction. B) Differential gene expression testing was performed for all pairwise combinations of conditions. Genes with a log_2_ fold change (Log_2_FC) greater than 1 or less than -1, and a false discovery rate (FDR) adjusted p-value less than 0.05 were defined as differentially expressed genes (DEGs). Collectively, 2374 genes were differentially expressed in at least one comparison between conditions. Expression of these genes is shown as a heatmap of log2 transformed FPKM values averaged across replicates in the glucose (GLUC), sucrose (SUC), glucose-deficient (DEF) and glucose-starved (STV) conditions. Expression is represented as row-scaled Z-scores. The dendrogram indicates hierarchical clustering of the dataset using Pearson correlation. C) Correlation of average gene FPKMs for cells shifted to glucose or sucrose conditions. D) Overlap of genes differentially expressed in glucose-deficient vs. glucose (yellow) and glucose-starved vs. glucose (blue). Upregulated (B) and downregulated (C) genes are shown. Size is proportional to number of genes in each subset. P-values were calculated based on the hypergeometric probability distribution. E) Enriched GO categories for the set of differentially expressed genes unique to the glucose-deficient vs glucose (DEF), glucose-starved vs glucose (STV) comparisons, or shared between both (SHARED). Degree of enrichment presented as -log_10_(adjusted P-value). Red categories are enriched in the upregulated population while blue categories are enriched in the downregulated population. GO categories of interest are shown in bold.


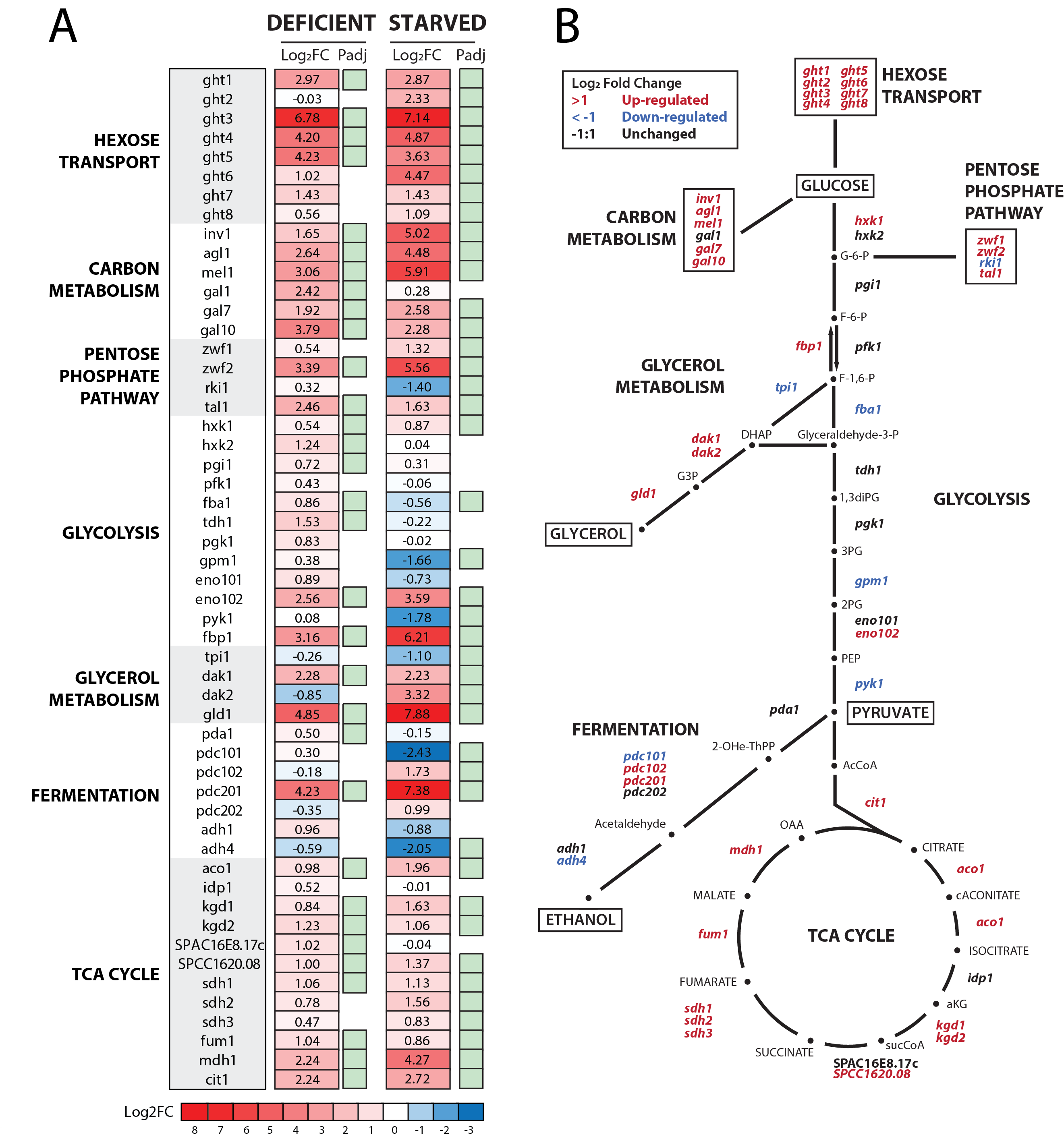


**Figure S3. Primary carbon metabolism pathways are upregulated in the absence of glucose.** A) Heatmap of log2 fold expression changes (Log_2_FC) for selected genes involved in carbon metabolism pathways for the glucose-deficient (DEFICIENT) or glucose-starved (STARVED) conditions compared to glucose. Green boxes indicate gene expression changes significant at an FDR < 0.05 (Padj). Degree of red/blue colouring indicates degree of up/downregulation respectively. B) Pathway diagram of carbon metabolism genes from A) coloured according to their differential expression in the glucose-starved condition vs glucose (see key). Significant upregulation of almost all genes functioning in primary carbon metabolism pathways: carbohydrate metabolism, hexose uptake, the pentose phosphate pathway, glycerol metabolism, fermentation and the TCA cycle is apparent suggesting that the glucose-deficient/glucose-starved conditions elicit significant changes to these processes in wild type *S. pombe* cells (Additional file 1: Figure S2A). Interestingly, just two glycolysis/gluconeogenesis genes, *fbp1^+^* and *eno102^+^* were more strongly induced in the glucose-starved condition relative to the glucose-deficient condition, while most others were repressed compared to the glucose-deficient condition.


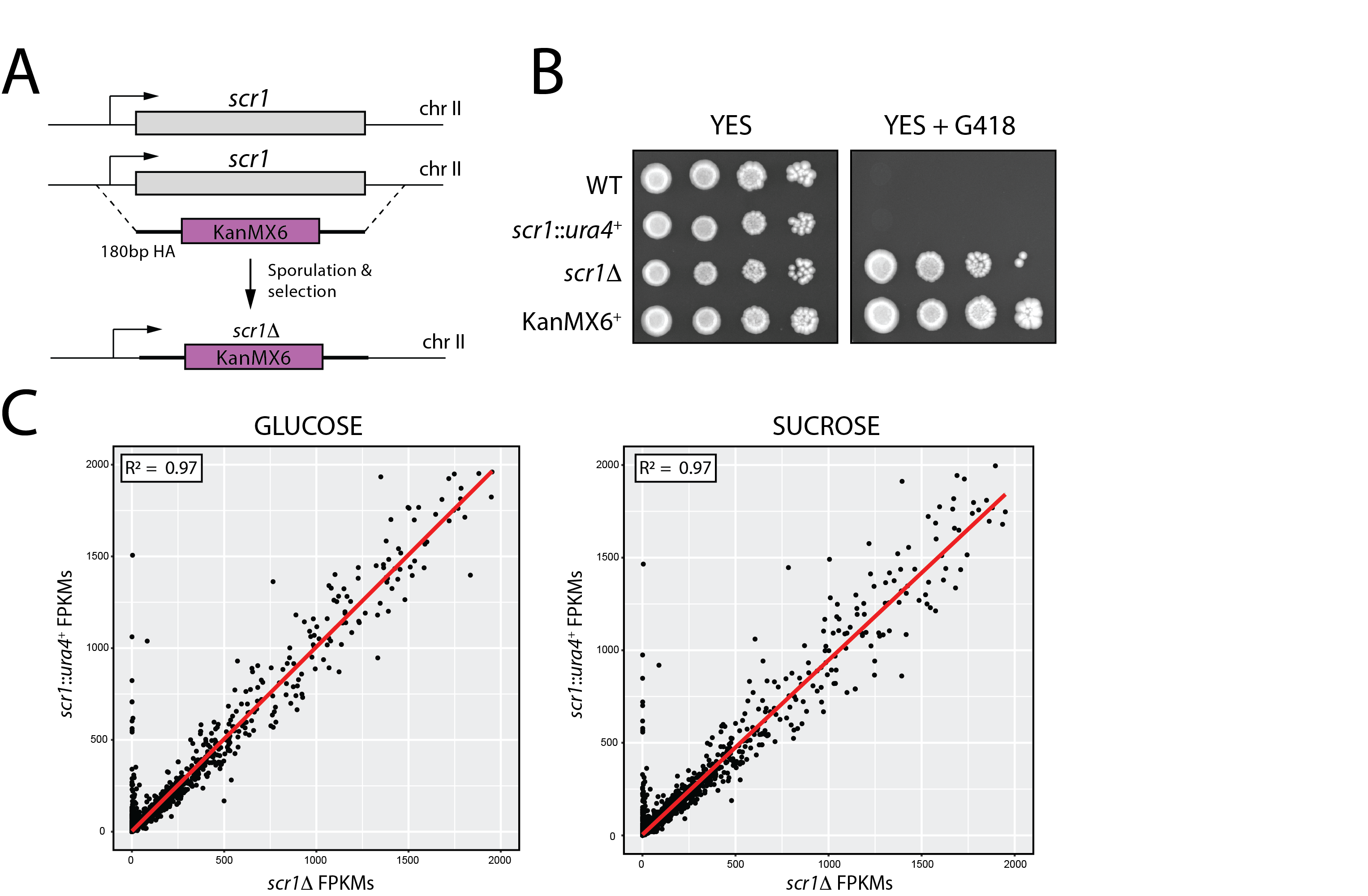


**Figure S4. *scr1*::*ura4*^+^ disruption is a faithful representation of *scr1*^-^ loss of function.** A) Tenfold dilutions of wild type (WT, 972*h*^-^), *scr1*::*ura4*^+^ and *scr1*Δ cells were spotted onto YES and YES + G418 and imaged after 48hr growth at 32°C. A strain carrying the KanMX6^+^ cassette at an unrelated locus (*rmt3*^+^) is shown as a positive control for G418 resistance. No differences between *scr1*::*ura4*^+^ and *scr1*Δ cells are apparent. B) Regression analysis of *scr1*::*ura4*^+^ and *scr1*Δ RNA-seq performed using FPKM values averaged from two biological replicates of samples grown in glucose, sucrose, glucose-deficient or glucose-starved conditions. Correlation (Adj R^2^), Intercept, Slope and P- value (P) of the line of best fit (red line) are indicated above each plot. The *scr1*::*ura4*^+^ and *scr1*Δ RNA-seq are highly correlated (R^2^ > 0.95 in each condition).


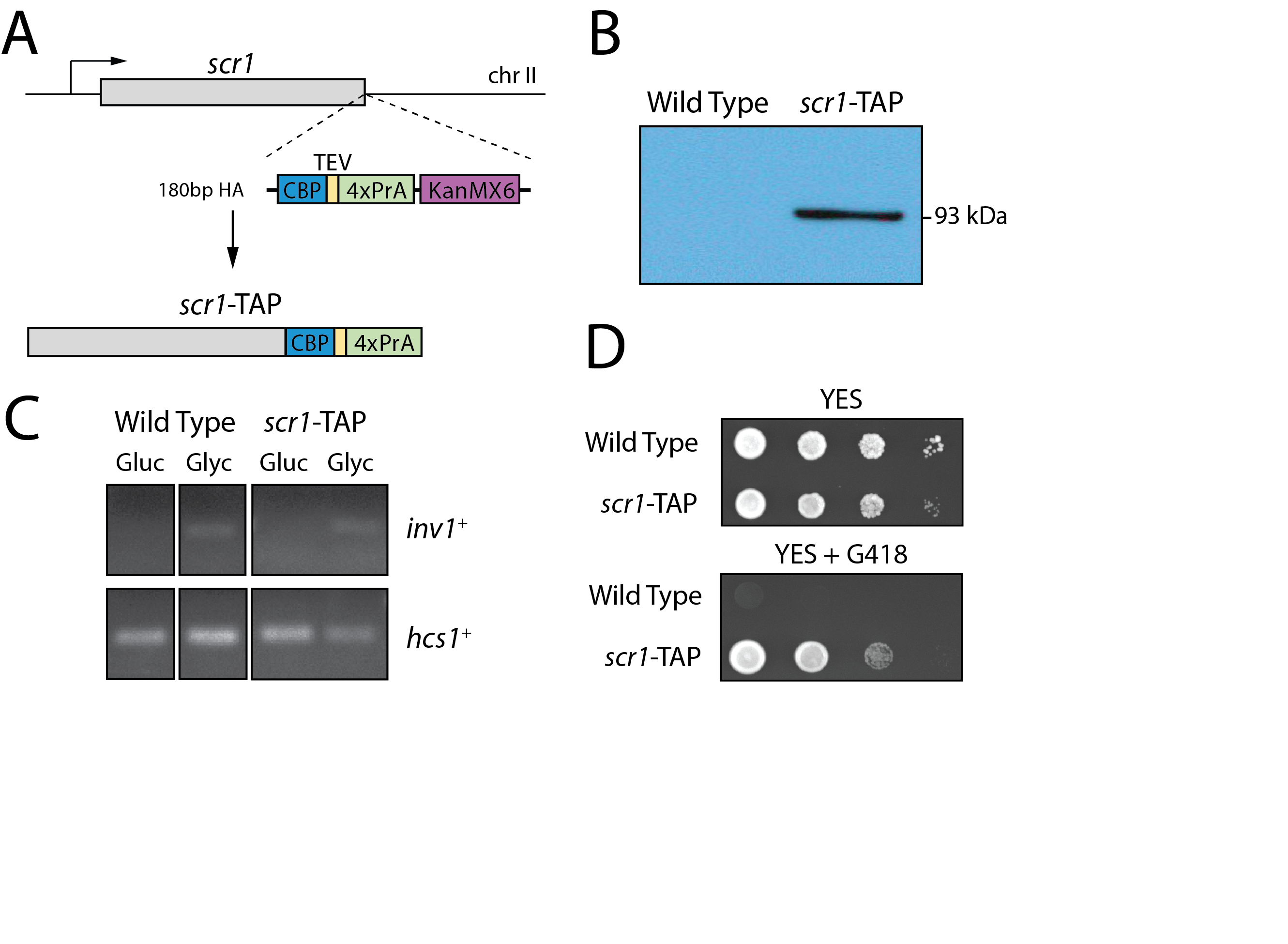


**Figure S5.** **Generation of a TAP-epitope tagged Scr1 expression *S. pombe* strain.** A) Schematic of the TAP-tag construct amplified from plasmid pFA6a-4X-CTAP (Materials and Methods) and its integration in-frame with the *scr1*^+^ CDS. 180bp regions of homology were used to target the construct for homologous recombination into the *S. pombe* genome. B) Western blot of wild type (972*h*^-^) and Scr1-TAP (D51) whole cell protein lysates using an α-Protein A-HRP antibody at 1:1000 dilution. Expected size of the Scr1-TAP fusion protein is indicated on right hand side of panel. C) Semi-quantitative RT-PCR of *inv1*^+^ and *hcs1*^+^ (loading control) for wild type (972*h*^-^) and Scr1-TAP (D51) strains grown in YES 3% (w/v) glucose and shifted after four hours to YES 3% (w/v) glucose (Gluc) or YES 3% (v/v) glycerol (Glyc). D) Spot dilution assays of wild type (972*h*^-^) and Scr1-TAP (D51) cells on YES and YES + G418. Results shown are representative of three independently generated Scr1-TAP expressing strains.


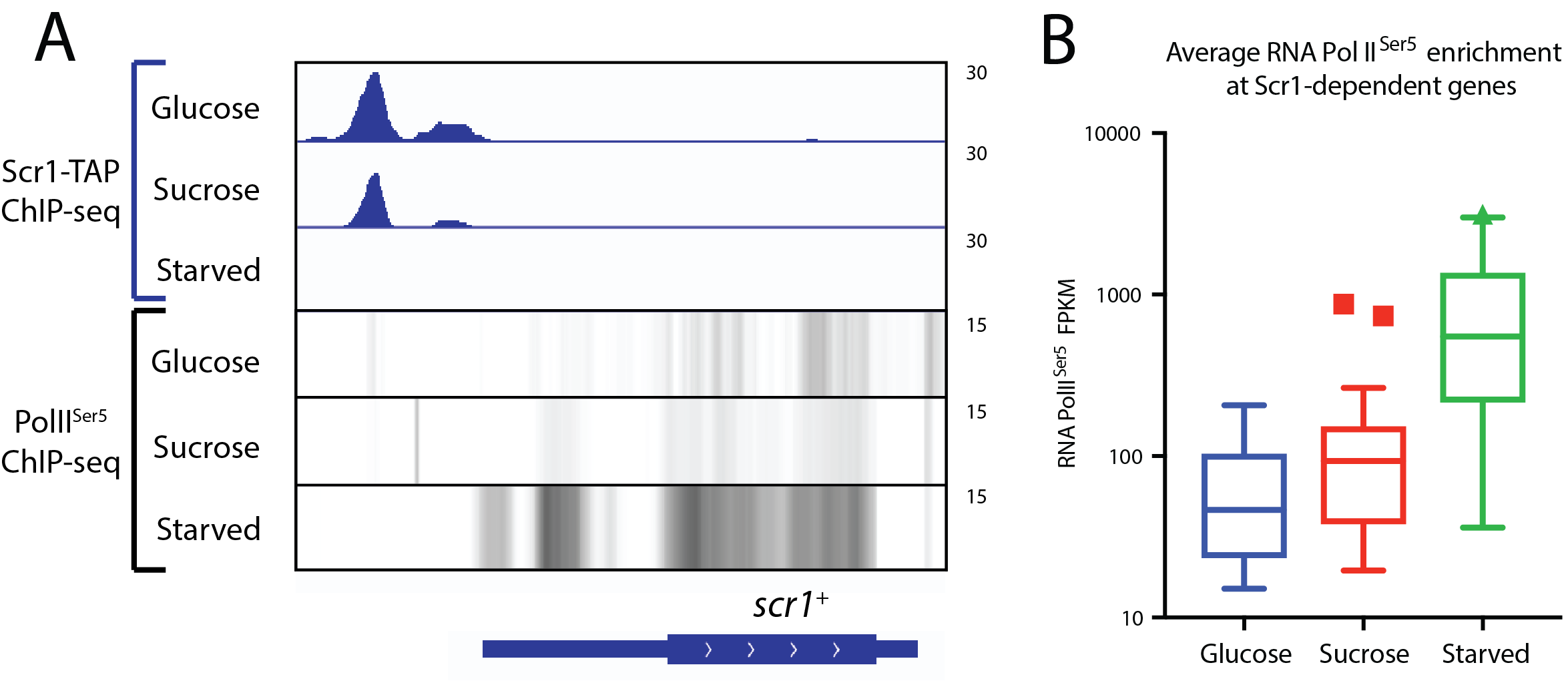


**Figure S6. Scr1 possesses autoregulatory capacity.** A) Genome browser visualization of Scr1 and RNA PolII^Ser5^ enrichment at the *scr1*^+^ locus. Scr1 is bound to the *scr1*^+^ promoter in glucose and sucrose conditions where it shows little RNA PolII^Ser5^ enrichment. Conversely Scr1 is absent in glucose-starved conditions (“Starved”) where RNA PolII^Ser5^ is highly enriched. This pattern extends to Scr1-dependent genes. B) shows Tukey style boxplots of average RNA PolII^Ser5^ enrichment (expressed as Fragments Per Kilobase per Million mapped reads, FPKM) in glucose, sucrose and glucose-starved (“Starved”) conditions for the 32 Scr1 dependent genes.


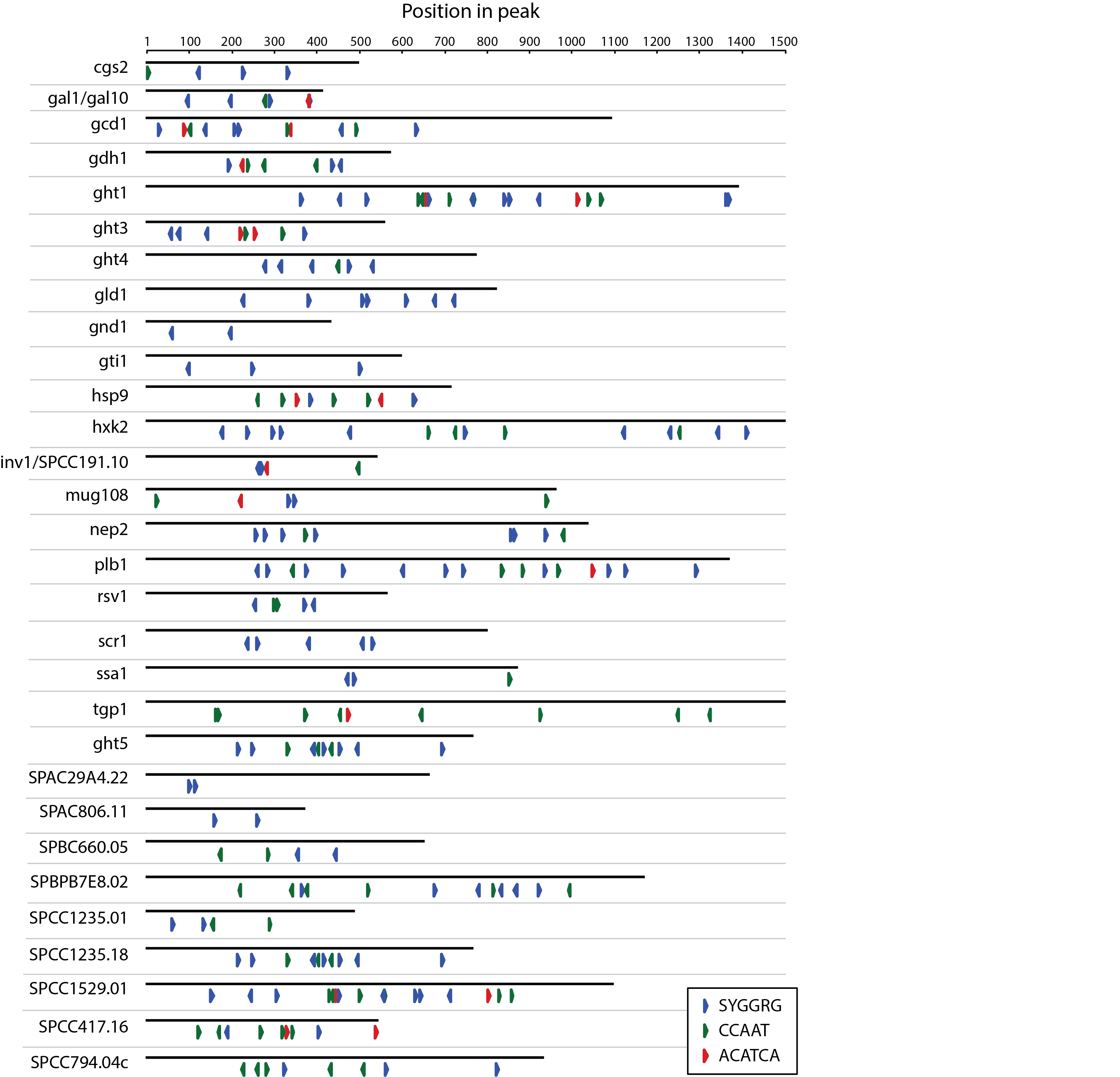


**Figure S7. The promoters of Scr1-dependent genes contain putative Scr1, HAP complex and ATF/CREB factor binding sites.** The regions of significant Scr1 enrichment identified in the promoter of 29 of the 31 Scr1-dependent genes were examined for the presence of SYGGRG (Mig1/CreA, blue), CCAATC (HAP complex, green) or ACATCA (ATF/CREB-like, red) motifs. Peak sequences are represented by black bars. Motifs on positive strand face right. Motifs on negative strand face left. *fbp1*^+^ and *ght5*^+^ are included as positive controls. Position in peak is indicated via the top scale in base pairs.


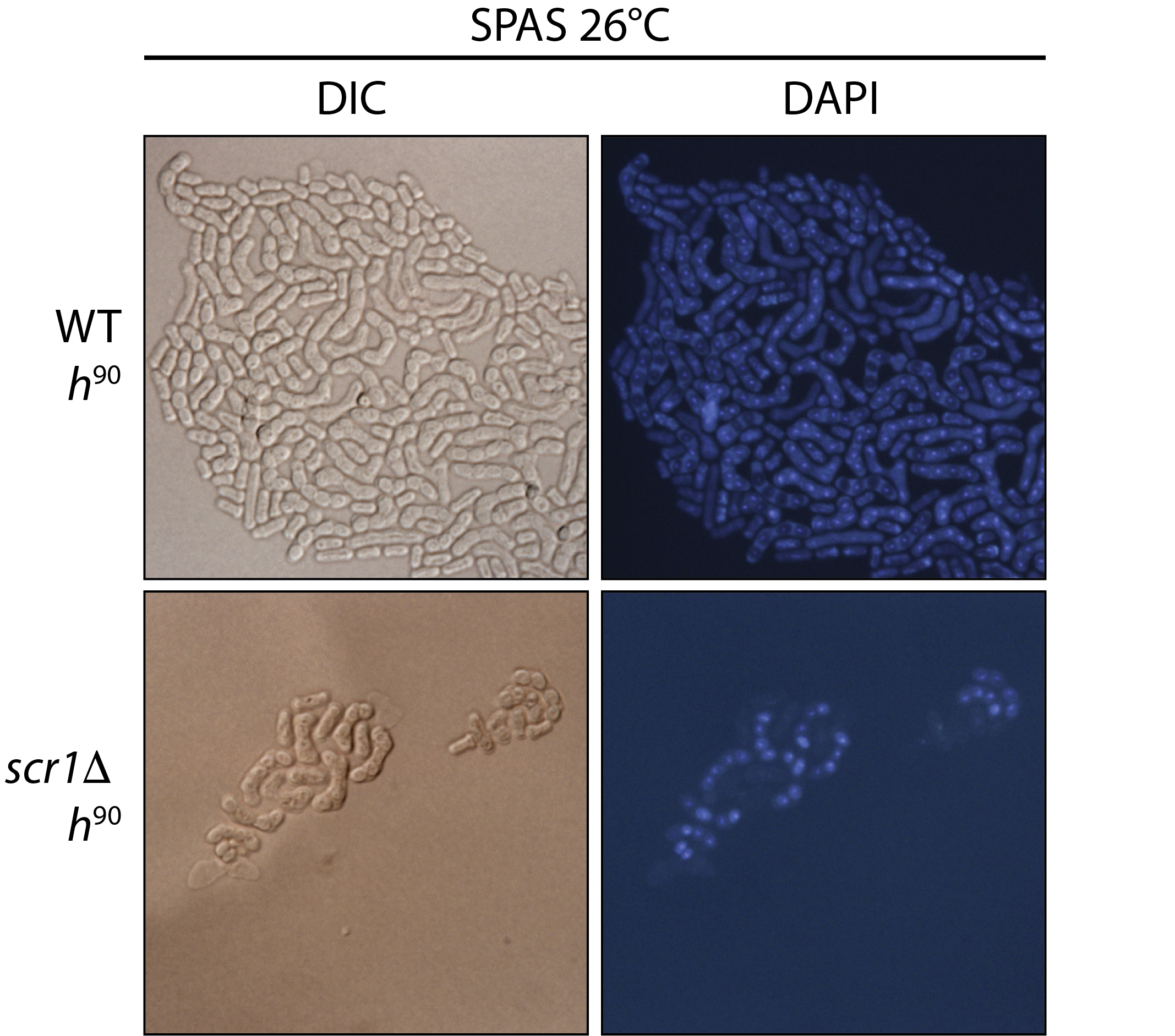


**Figure S8. *scr1*Δ *h*^90^ cells mate normally on SPAS medium at 26 degrees.** DIC and DAPI microscopy of homothallic wild type (WT *h*^90^) and *scr1*Δ *h*^90^ (D179/D181) *S. pombe* cells grown on SPAS at 26°C for 48 hours. Scale bars = 20µm.


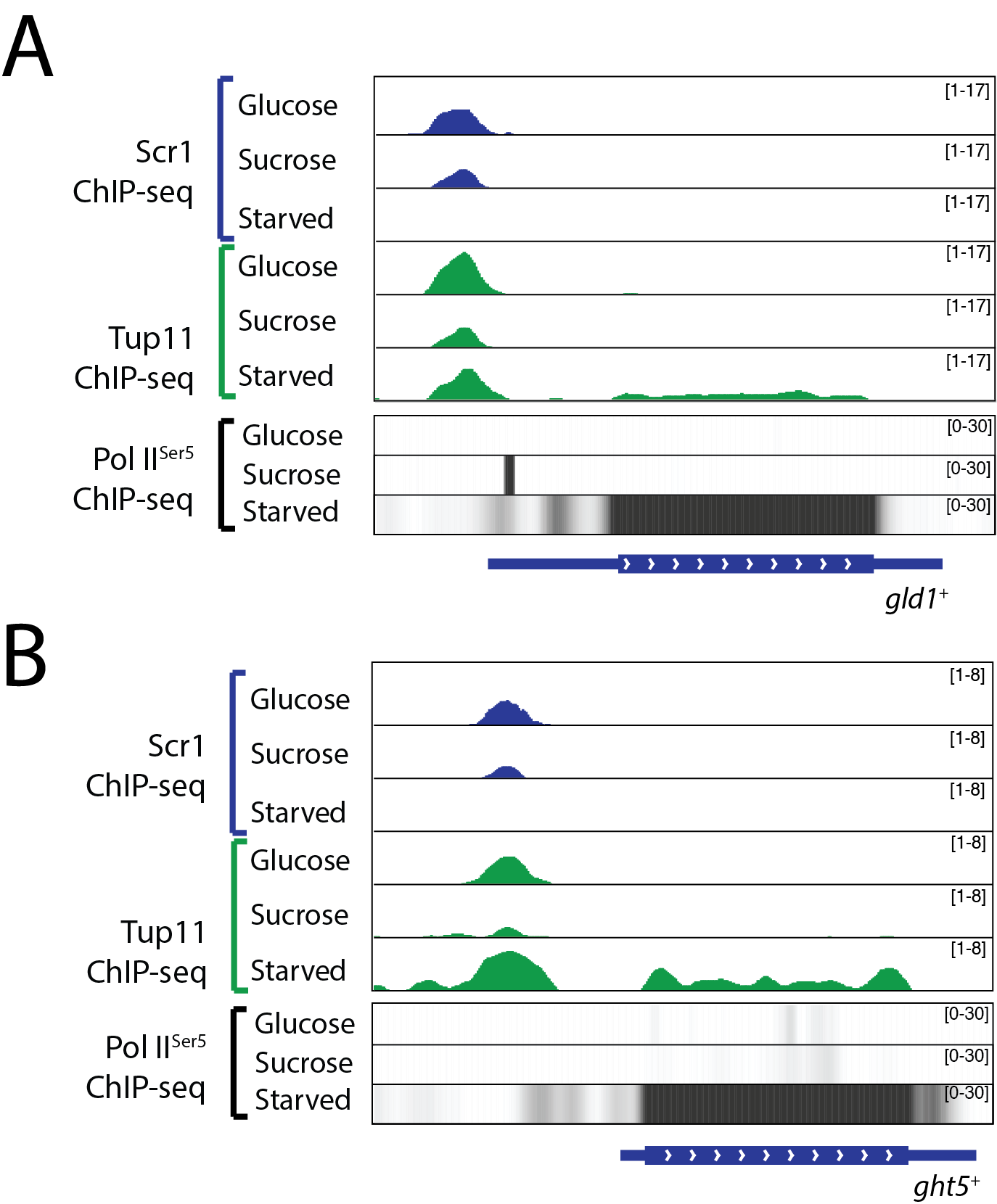


**Figure S9. Scr1 and Tup11 co-localise at the promoter of known Scr1 target genes.** Genome browser visualizations of Scr1, Tup11 and RNA PolII^Ser5^ at the *gld1*^+^ (A) and *ght5*^+^ (B) loci. Coverage tracks are corrected for background enrichment and normalized to reads per 1x reference genome coverage (1x RPGC). RNA PolII^Ser5^ enrichment is presented as a heatmap.


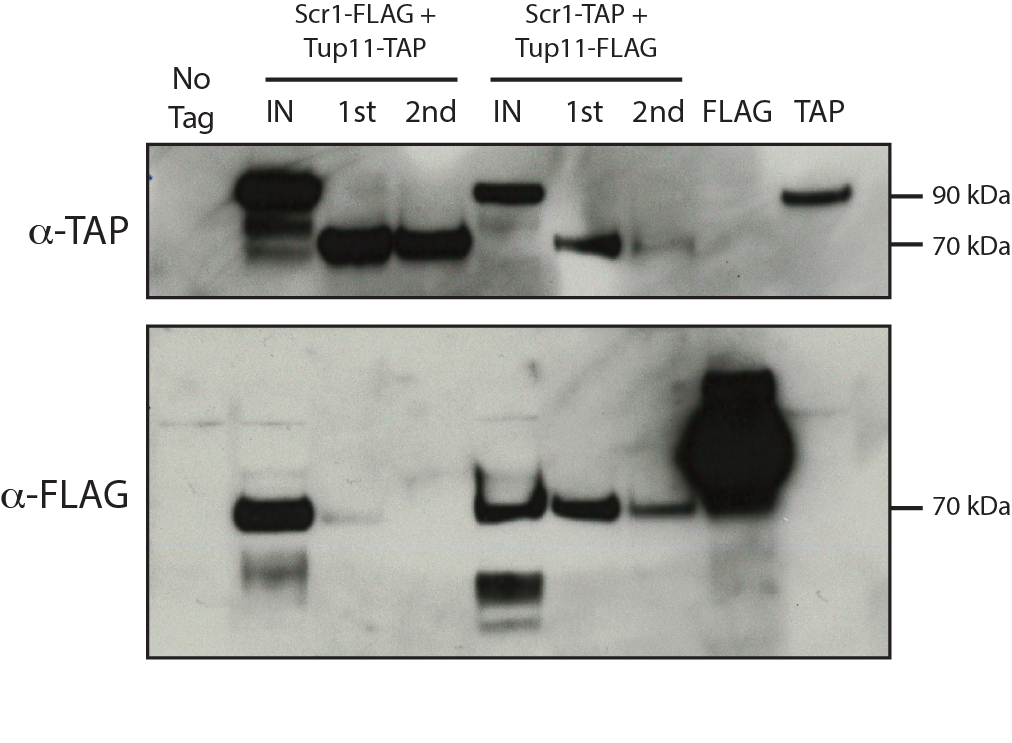


**Figure S10. Scr1 and the Tup/Ssn6 complex physically interact but do not form a stable stoichiometric protein complex.** Western blot of Scr1-FLAG in Tup11-TAP purified protein samples and Tup11-FLAG in Scr1-TAP purified protein samples. IN = input whole cell protein lysate. 1st = Protein eluted from primary affinity column following TEV cleavage. 2nd = Protein eluted from secondary affinity column following elution with EGTA. The α-TAP panel shows a western blot probed with anti-TAP monoclonal antibody (Pierce CAB1001) at 1:5000 dilution. The α-FLAG panel indicates a western blot probed with anti-FLAG M2 monoclonal antibody (CSIRO) at 1:5000 dilution. A wild type protein lysate is included as a negative control “No Tag”. Unrelated FLAG and TAP-tagged protein samples are included as positive controls (“FLAG” and “TAP” respectively). Sizes of proteins in 1st and 2nd samples in the α-TAP panel are smaller compared to the IN sample due to TEV cleavage of the Protein A moiety of the TAP tag prior to the second affinity purification step. Relative size markers are shown on the right-hand side of each panel. Blots shown are representative of two independent biological replicates.


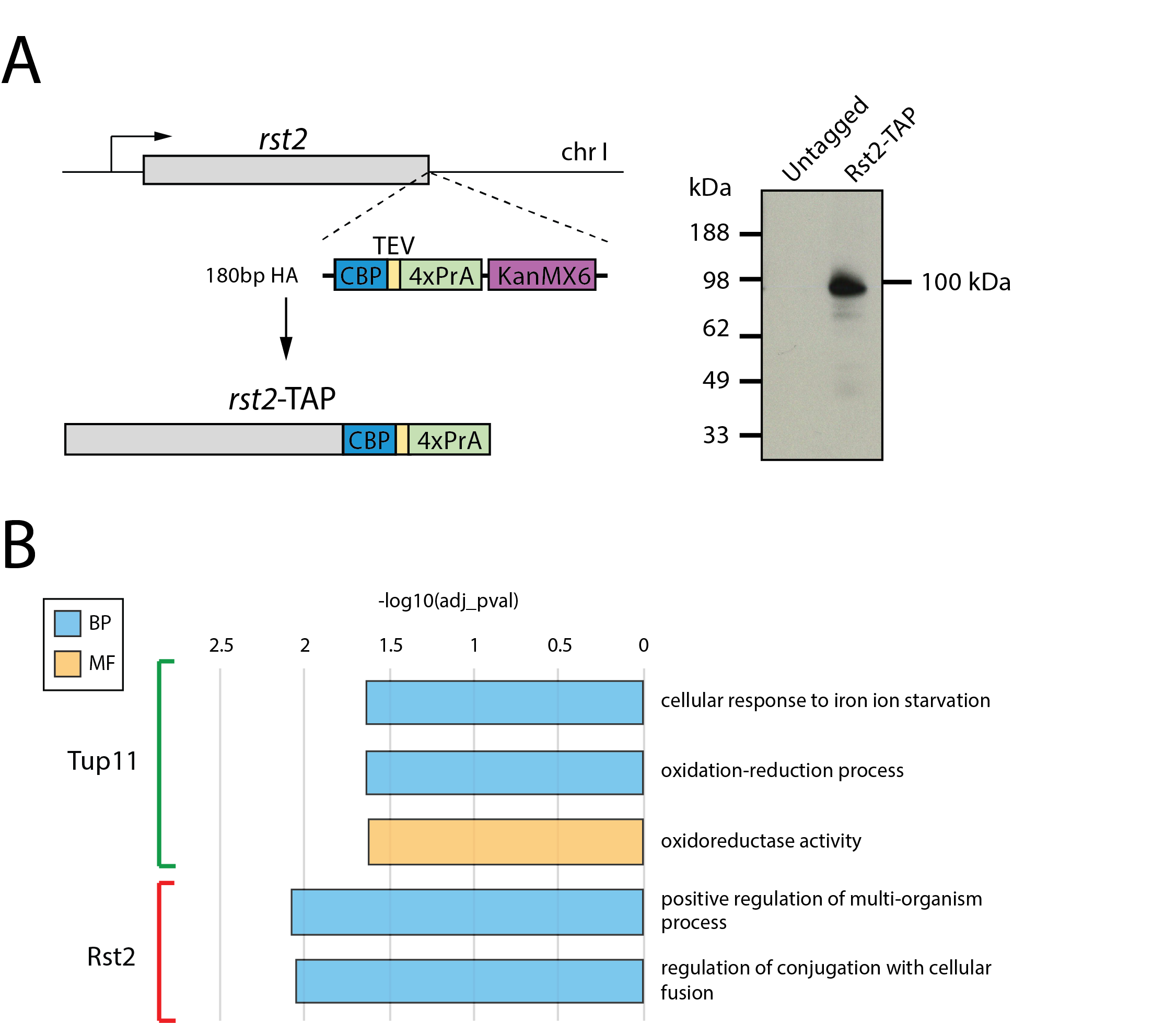


**Figure S11. GO Enrichment of Rst2 and Tup11 independent gene targets.** A) Schematic of the TAP-tag construct amplified from plasmid pFA6a-4X-CTAP (Materials and Methods) and its integration in-frame with the *rst2*^+^ CDS. 180bp regions of homology were used to target the construct for homologous recombination into the *S. pombe* genome. Right hand side panel shows a representative western blot of wild type (D1, “Untagged”) and Rst2-TAP (D101) whole cell protein lysates using an α-Protein A-HRP antibody at 1:1000 dilution. Expected size of the Rst2-TAP fusion protein is indicated on right hand side of panel. B) Enriched GO categories for the loci bound uniquely by Tup11 or Rst2 in the glucose-starved condition. BP = Biological process, MF = molecular function.


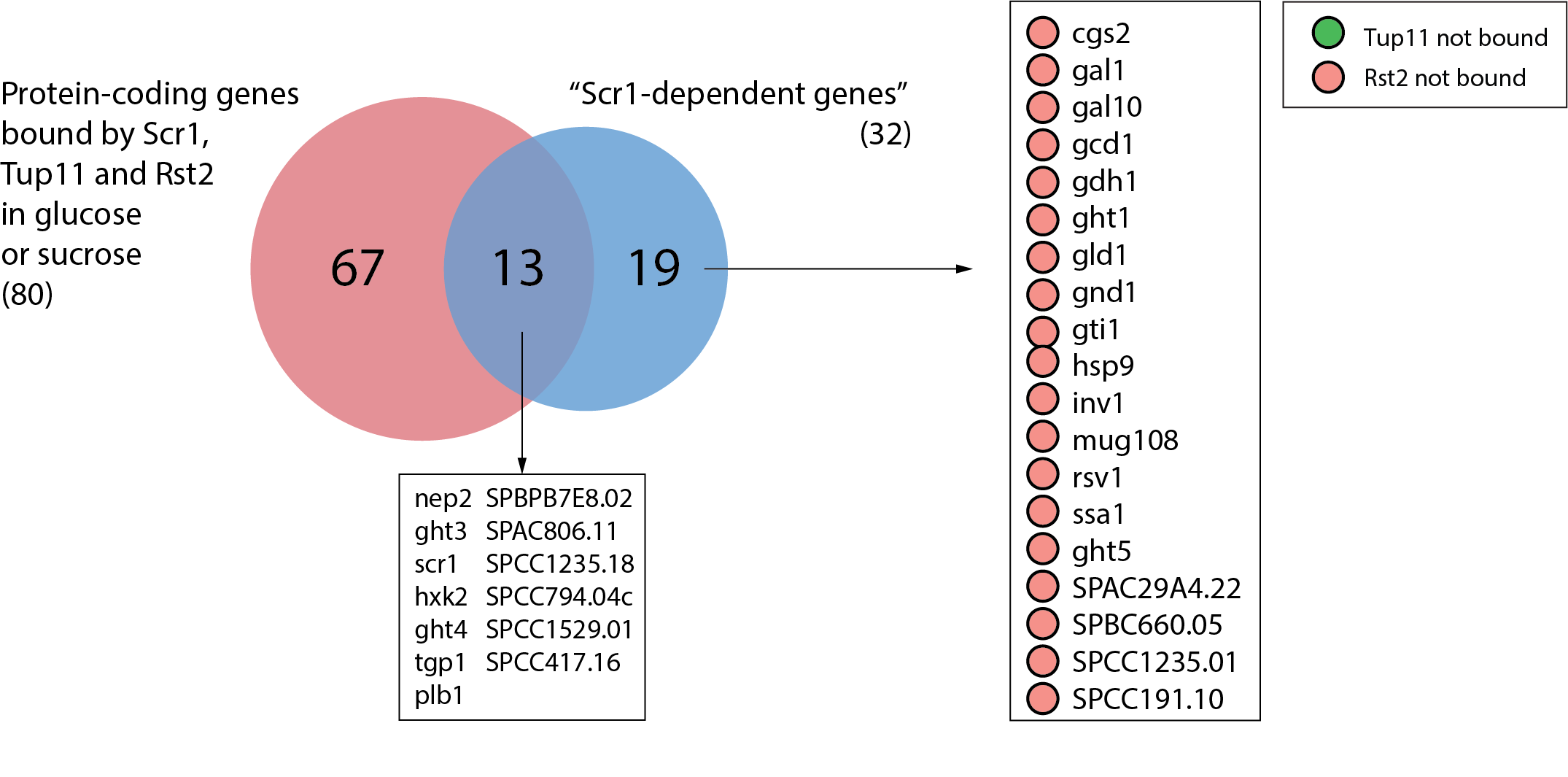


**Figure S12. Overlap of protein coding genes bound by Scr1, Tup11 and Rst2 in glucose or sucrose and the “Scr1-dependent” gene set.** Venn diagram shows overlap between the two datasets with genes comprising the intersection shown. For the “Scr1-dependent genes” unique fraction, the coloured circles represent which factor (i.e. Tup11 – green, or Rst2 – red) was not bound in glucose or sucrose conditions.
